# Light Penetration, Dissolved Oxygen, and Riparian Zone Extent Are Associated with the Structure, Gene Expression, and Lipid Production of Riverbed Microbial Communities

**DOI:** 10.3390/microorganisms14102406

**Published:** 2026-10-21

**Authors:** Vijay Shankar, Oleg Paliy

**Affiliations:** 1 Department of Biochemistry and Molecular Biology, Boonshoft School of Medicine, Wright State University, Dayton, OH 45435, USA; vshanka@clemson.edu; 2 Institute for Human Genetics, Clemson University, Greenwood, SC 29646, USA

**Keywords:** riparian zone, benthic microbiota, sediment microbiome, fatty acids, metatranscriptomics, algae, agricultural runoff, eutrophication

## Abstract

We analyzed riverbed microbiota structure, gene expression, and fatty acid production across several sites along Little Miami River in Ohio, United States. The sites varied in stream width, shading, surrounding land use, and the size of the riparian zone. Within each site, we observed strong concordance among microbial community composition, functional gene expression, and fatty acid signature. All microbial communities were dominated by the members of phylum Proteobacteria, class Planctomycetacia, and by algal and cyanobacterial cells. The measured concentrations of chlorophyll *a*, dissolved oxygen, and soluble phosphorus were the main environmental determinants of microbial community structure. These were in turn dependent on the level of penetrating sunlight and, to a lesser degree, on the width of the riparian zone. The level of sunlight affected the expression of light-harvesting complexes, methanogenesis genes, anaerobic respiratory reductases and fermentation pathways, as well as functions of denitrification, ammonification, and sulfate reduction. The expression of phosphorus scavenging and antibiotic resistance genes was highest at the site lacking a riparian zone and surrounded by farmland. Overall, our findings demonstrate that riverbed microbiota exhibit consistent structural and functional responses to interacting geochemical and anthropogenic gradients. Our data also provide support for the hypothesis that phototrophic microorganisms act as central organizers of benthic community metabolism.

## 1. Introduction

Riverbed sediments host densely packed, vertically stratified microbiomes that drive carbon, nitrogen, sulfur, and metal cycling across significant redox and light gradients [1]. Because oxygen penetrates settled river sediment only to the depths of a few millimeters, distinct geochemical zones develop that support photosynthesis, nitrification, denitrification, sulfate reduction, methanogenesis, and methanotrophy [2]. Microbial mats and particle-associated biofilms consequently comprise oxygenic phototrophs, including diatoms and cyanobacteria near the sediment surface, anoxygenic phototrophs such as purple and green sulfur bacteria in deeper low-oxygen layers, and heterotrophs that use nitrate, metal oxides, or sulfate as terminal electron acceptors (reviewed in [3]). Light availability and sediment grain size regulate phototroph distribution and metabolic layering [4], while vertical physicochemical gradients structure microbial diversity and facilitate nutrient and chemical cycling among community members [5,6,7]. Organic matter breakdown, both from natural sources (e.g., plant leaves falling into the river) and from surrounding agricultural activities, also has a major effect on the functioning of this ecosystem [8].

In addition to the increased organic matter, agricultural runoff also delivers inorganic nutrients (N, P), sediments, pesticides, and antibiotics into river systems [9,10,11]. Several meta-analyses linked the agricultural use of surrounding land to river biodiversity loss, turbidity increases, eutrophication risk, and altered microbial community structure [12,13]. Increasing inputs of nitrogen, phosphorus, and agrochemicals often favor copiotrophic taxa and stimulate the expression of genes involved in nutrient assimilation and xenobiotic degradation [10,14]. Elevated nutrient loading can also suppress oligotrophic and redox-specialist microbes, altering pathways related to denitrification, methanogenesis, and sulfur cycling [10,15]. The elevated nitrate and phosphorus from farm crop landscapes also fuel algal blooms [16], and algal biomass acts as a top-down driver of bacterial traits [7]. Subsequent nutrient decomposition and sediment oxygen use depress dissolved oxygen and modify microbial community structure and function [17].

The effects of agricultural land use on river systems are also influenced by the presence, continuity, and width of riparian zones. Vegetated riparian buffers slow overland flow, trap sediments, and intercept nutrient- and pesticide-laden runoff before it enters the river channel, thereby reducing nutrient delivery and dampening eutrophication pressure on benthic phototrophic and heterotrophic assemblages [18]. Riparian vegetation also stabilizes banks, regulates stream temperature through shading, and maintains hydrological connectivity—processes that maintain microenvironmental heterogeneity supporting diverse sediment microbiota [19]. Wooded riparian buffers can further reduce nutrient and sediment loading (further reviewed in [20]). Intact riparian zones were also shown to promote the expression of genes linked to heterotrophy, stress tolerance, and biogeochemical resilience [21,22]. Together, agricultural pressures and riparian-zone integrity contribute to spatial variation in riverbed microbial community structure and transcriptional activity.

To provide further insight into these relationships, we analyzed the structure, gene expression, and fatty acid production of riverbed microbial communities from the benthic samples collected at several locations of the Little Miami River in Ohio, United States. The river locations were chosen to provide multiple gradients in the available light penetration, agricultural land proximity, and riparian zone width, thus allowing us to test the associations of these variables with the benthic microbiota.

## 2. Materials and Methods

### 2.1. Study Sites 

**Figure 1:**
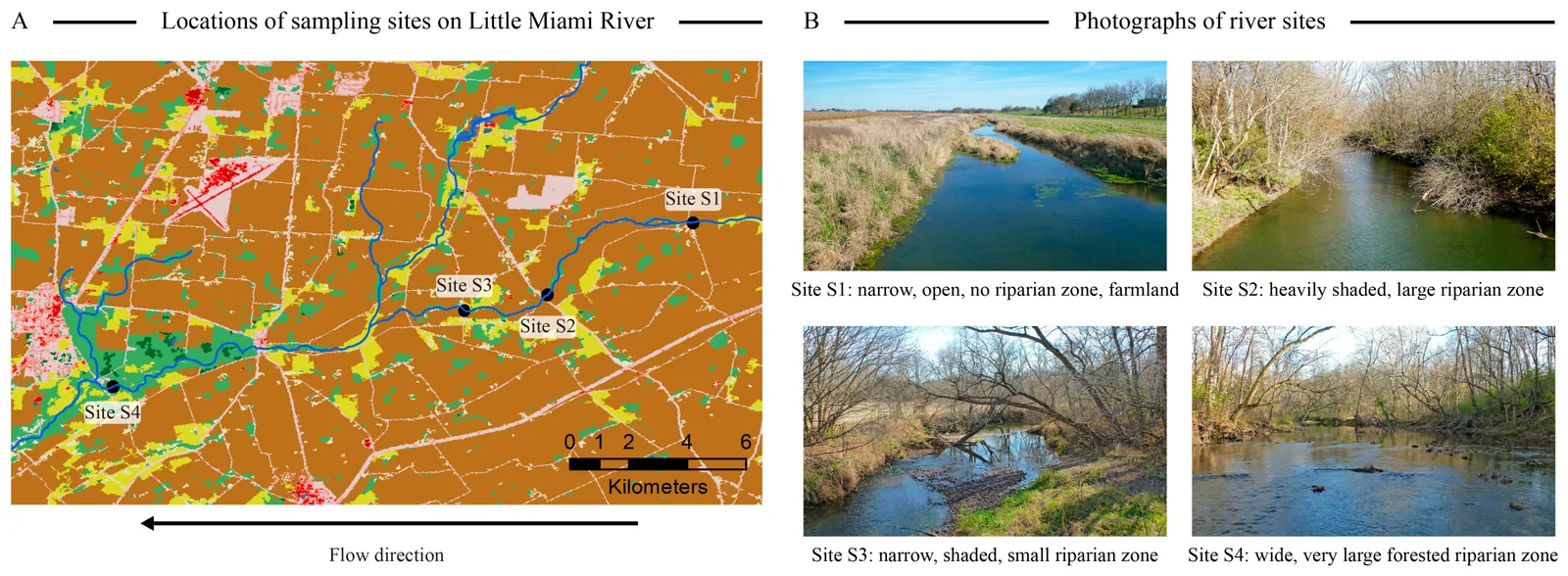
Map (**A**) and photos (**B**) of sampling locations along the Little Miami River. In panel (**A**), the map shows sampling locations (black circles) and land use as provided in the National Land Cover Database (2011). Approximate coordinates for site S1: 39°49′58.6″ N 83°41′39.7″ W; site S4: 39°46′58.4″ N, 83°52′15.0″ W. Land use categories: crop agriculture (brown), pasture (yellow), forest (green), developed (red), water (blue). The map and photographs were provided by Dr. Hannah Fazekas (Wright State University, USA).

The sediment microbial communities were characterized at four sites in the Little Miami River during the late fall season (chosen because of the reduced fertilizer use during that period; Figure 1A). Sites were selected to capture the natural variation in terrestrial organic carbon subsidies and attached algal biomass (Figure 1B). Site S1 is a headwater stream draining agricultural land that has no forested riparian area. Site S2 has a substantial forested riparian area that shades the entire stream during the growing season, while site S3 has a small riparian buffer strip that only partially extends over the stream. The most extensive forested riparian area occurs at site S4 where the river has widened to the point where there is direct sunlight during the day.

A number of measured environmental variables were available for the above-described sites, and this dataset was kindly provided by Dr. Hannah Fazekas (Wright State University) from her previous study [23]. The environmental variables included the river width (WDTH), the riparian zone width (RZ), thalweg temperature (TEMP), pH, conductivity (COND), dissolved oxygen (DO), and the percent light reaching stream sediment at each site. Water samples were also previously collected and analyzed for soluble reactive phosphorus (SRP, *n* = 3 technical replicates), total suspended solids (TSS, *n* = 3 technical replicates), and chlorophyll *a* analyses (CHLR, *n* = 3 technical replicates) at each site as described [23]. The values of these variables at each site are provided in Table 1 for reference.

### 2.2. River Sediment Collection

Eight sediment samples from each site were previously collected using sterile 50 mL centrifuge tubes [23]. Samples were collected approximately at equal intervals across two transects perpendicular to the streamflow and immediately placed on ice for transport to the laboratory. Gene expression alterations were stopped by the addition of 1/10 volume of 95% ethanol/5% acidic phenol mixture [24]. Each tube was centrifuged at 4500 rpm and overlying water was decanted and discarded. For each site, these bulk sediment samples were homogenized together and the mixtures were aliquoted into microcentrifuge tubes. Tubes were frozen and kept at −80 °C until required.

### 2.3. Isolation of Genomic DNA and High-Throughput DNA Sequencing

Total genomic DNA (gDNA) was isolated from approximately 150 mg of sediment material using PowerSoil DNA Isolation Kit (Qiagen, Germantown, MD, USA). Isolated bacterial genomic DNA was amplified in a PCR reaction targeting V4 hypervariable region of the 16S rRNA gene using degenerate forward (16S rRNA gene complementary sequence GCCAGCMGCCGCGG) and reverse (complementary sequence GGACTACHVGGGTWTCTAAT) primers [25]. The forward primers contained a 6-nucleotide barcode to permit sample pooling. PCR reaction consisted of 10 linear and 25 exponential cycles in order to reduce PCR amplification biases [25].

Generated amplicons were sequenced on the Ion Torrent Personal Genome Machine (PGM) using Ion 318 v2 chip (Thermo Fisher Scientific, Waltham, MA, USA) as described previously [26]. An average of 50,663 ± 17,585 reads was obtained per sample. Low-quality (average Q < 25) and short (<180 nt) reads were removed, and remaining high-quality reads were analysed in QIIME v1.8 as we described previously [25]. Microbial phylotypes were defined by clustering at 97% sequence similarity, and taxon annotation was based on the Ribosomal Database Project Classifier v2.11 and RDP 16S rRNA training set 19 (Michigan State University, East Lansing, MI, USA). All taxon counts were divided by the calculated average 16S rRNA gene copy number for that taxon in order to derive cell abundances [27], and these cell counts were then subsampled to the lowest number. This final dataset was used for all multivariate analyses. Cumulative table of genus-level cell counts in all samples is provided in Supplementary Table S1.

### 2.4. Isolation and Sequencing of Metatranscriptomes

Total microbial RNA was isolated using the PowerSoil RNA Isolation Kit (Qiagen, Germantown, MD, USA) according to the manufacturer’s protocol. The quality and fragment profile of the extracted RNA were assessed using 1% agarose gel and Bioanalyzer 2100 (Agilent, Santa Clara, CA, USA) with RNA Nano Chip. Ribosomal RNA was depleted using the RiboMinus Transcriptome Isolation Kit (Thermo Fisher Scientific, Waltham, MA, USA) with the RiboMinus bacterial module as well as custom biotinylated probes designed to remove Eukaryotic rRNA fragments. The Clean & Concentrator Kit (Zymo Research, Irvine, CA, USA) was used to increase the quality and concentration of the rRNA-depleted RNA samples. cDNA libraries were prepared using Ion RNA-Seq kit v2 (Thermo Fisher Scientific, Waltham, MA, USA), and size selection of cDNA libraries was performed on Pippin Prep (Sage Science, Beverly, MA, USA) with a fragment window of 100–360 base pairs. The high-quality, size-selected cDNA libraries were sequenced on an Ion Torrent PGM using the 318 v2 chip (Thermo Fisher Scientific, Waltham, MA, USA) and 500 sequencing flow cycles. An average of 4.5 ± 0.6 million raw sequences (minimum 4.0, maximum 5.3 million) were obtained per sample. All high-throughput sequencing data are available from the Sequence Read Archive, accession number PRJNA1458852.

Sequenced metatranscriptomic data were analyzed and annotated using the MetaTrans comprehensive analysis pipeline [28]. Briefly, post-sequencing rRNA filtering was performed using SortMeRNA v1.9 [29]. High-quality, rRNA-depleted sequences were scanned for open reading frames to identify protein-coding genes using FragGeneScan v1.17 [30]. Amino acid sequences from the protein-coding genes were clustered at 90% sequence identity using CD-HIT v4.6.1 [31]. Cluster representatives from CD-HIT were annotated using Diamond’s BLASTP v0.7.9 algorithm [32] and M5nr’s subsystem-based amino acid database [33]. Normalization of gene expression counts was performed at the lowest (FIG ID) level of the database using *voom* transformation command from the *limma* R v4.0 package [34]. Voom algorithm estimates the mean-variance relationships of the log-counts and generates a precision weight for each observation. The normalized counts were summed up at each subsequent higher hierarchical level (SEED L1 through L3). Cumulative table of L3-level expressed functions in all samples is provided in Supplementary Table S2.

### 2.5. Fatty Acid Measurements 

Fatty acid measurements were conducted for the profiled sediments in a prior study [23] via gas chromatography–mass spectrometry (GC-MS) analysis of fatty acid esters, and the dataset of raw fatty acid abundances was kindly provided by Dr. Hannah Fazekas (Wright State University). Fatty acid abundances were calculated by dividing individual markers by total sediment mass. We combined known fatty acids into the groups for prokaryotes, fungi, and algae to obtain relative abundance and biomass of these groups [35]. Algal and cyanobacterial markers included: 16:2ω4/6, 16:3ω3, 16:4ω3, 18:3ω3, 18:4ω3, 20:2ω6, 20:3ω3/6, 20:4ω6, 20:5ω3, 22:4ω6, 22:5ω3/6, 22:6ω3. Fungal markers included 16:1ω5 and 18:2ω6. Prokaryotic markers included i-14:0, a-15:0, i-15:0, 15:1ω7, i-16:0, 16:1ω6/7/8/9, 16:2ω7, a-17:0, i-17:0, 18:1ω7/9, 18:3ω6, 20:1ω9, 22:1ω9 [35]. Cumulative table of measured fatty acids in all samples is provided in Supplementary Table S3 for reference.

### 2.6. Statistical Data Analyses 

Statistical procedures were carried out in R v4.0, SPSS v19 (SPSS, Inc., Chicago, IL, USA), and MATLAB 2015 (The Mathworks, Inc., Natick, MA, USA) using approaches that we described earlier [36]. To statistically estimate the distribution of environmental variables, ANOVA algorithm was employed with Tukey’s Honestly Significant Difference post hoc analysis. Multivariate ordination algorithms included principal components analysis (PCA), weighted UniFrac distance-based principal coordinates analysis (PCoA), distance-based redundancy analysis (dbRDA), and Co-Inertia analysis [36,37]. Because the number of environmental variables, which served as explanatory variables in our constrained ordination analyses, was significantly higher than the number of sites, factor analysis (FcA) was used to reduce the dimensionality of the explanatory variable dataset. Two factors were sufficient to capture the vast majority (93%) of the variability in the explanatory variable dataset. UniFrac distance-based principal response curves (UF-dbPRC) [37] were generated to interrogate the changes in microbiota composition along each factor. dbPRC analysis is a statistical approach that isolates and evaluates the relationship between a single explanatory variable (for example, either FcA factor 1 or factor 2) and the response variable dataset (for example, class-level microbial abundances among sites) among objects (sites). The sites are ordered according to the values of the evaluated explanatory variable, and the deviation of the response variable set from the baseline site is calculated.

## 3. Results

### 3.1. Characteristics of the Little Miami River Sampling Sites

Little Miami River is a class I tributary located in Ohio, USA, which flows through diverse landscapes as shown in Figure 1A. Four river sites were chosen that differed in several parameters (see Table 1). The percent of total sunlight reaching the stream’s riverbed varied significantly among the sites (S1: 88%, S2: 12%, S3: 24%, and S4: 54%, see Figure 1B, ANOVA F_3,14_ = 71.92, *p* < 0.001). Nine environmental variables were measured in addition to the light penetration as shown in Table 1. Among these, the width of the stream and of the riparian zone, the amount of chlorophyll *a*, and the concentration of soluble reactive phosphorus (SRP) varied noticeably among the sites. For example, mean SRP ranged from 0.48 ± 0.19 to 1.12 ± 0.18 mg L^−1^, and the most upstream site S1 had significantly higher SRP concentrations than the other sites as determined by the post hoc Tukey’s HSD test (ANOVA, F_3,14_ = 37.45, *p* < 0.05). The measured SRP amount was inversely proportional to the width of the riparian zone (Spearman correlation R_s_ = −1.00, note the small *n* = 4 potentially inflating the obtained correlation value). The amount of chlorophyll *a* was proportional to the percent of penetrating sunlight (R_s_ = 1.00).

### 3.2. Diverse Microbial Communities Are Found in Stream Sediment

**Figure 2:**
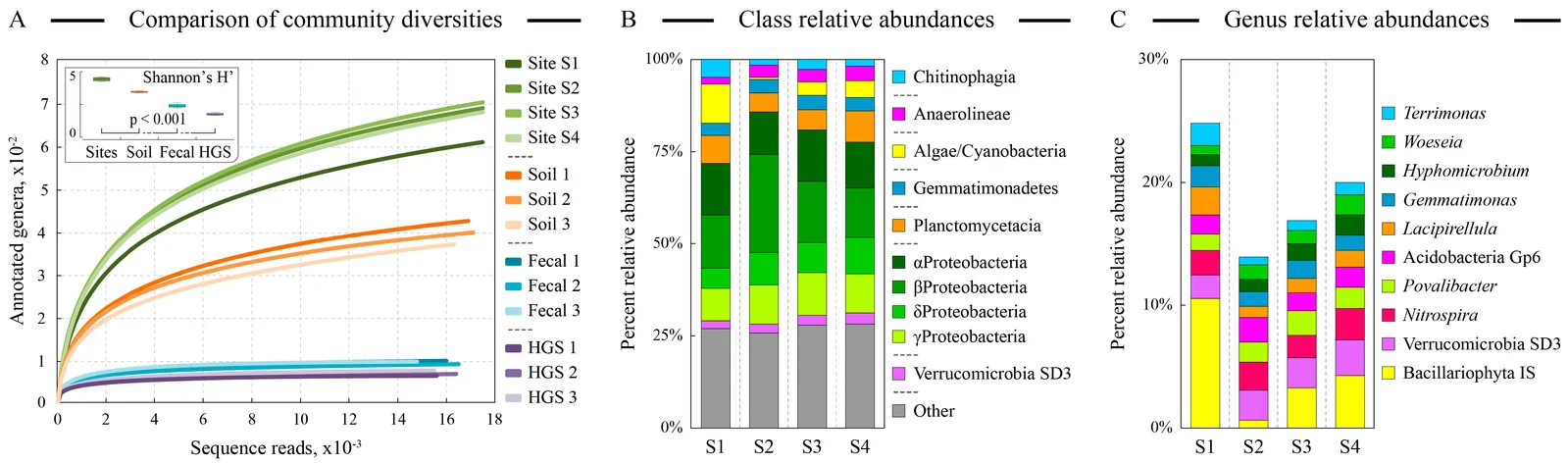
Diversity and relative composition of microbial communities. Rarefaction curves demonstrating the dependence of the total number of annotated genera on the number of obtained sequence reads are shown in (**A**). For comparison purposes, samples taken from wetland soil, human stool, and the in vitro human gut simulator (HGS) were profiled in addition to the four river sites. The inset shows the boxplots for the calculated Shannon’s H’ diversity indices among the samples of each type. Relative abundances of the top ten abundant classes and top ten abundant genera are shown in (**B**) and (**C**), respectively. The classes were ordered based on their phylum assignment, and the genera were ordered based on the average abundance across four sites. Where possible, genus colors match the corresponding class.

Utilizing 16S rRNA gene amplicon sequencing, we profiled the composition of microbial communities in the riverbed sediment of each site. Each community was highly diverse, with between 600 and 700 different genera and between 5900 and 7000 phylotypes detected at each site. We compared the number of annotated genera to those found in wetland soil, human fecal samples, and in vitro human gut simulator (HGS) system using comparable sample processing, sequencing, number of reads, and analytical pipeline. As shown by the rarefaction curves in Figure 2A, riverbed sediment communities were noticeably more diverse than those from other samples, based both on the number of annotated genera and on the calculated Shannon’s H’ index (*p* < 0.001 based on the 2-tailed *t*-test).

Overall, the microbial communities of sites S3 and S4 were the most similar among the sampled locations based on the beta diversity calculations of the phylogenetic weighted UniFrac distance (wUF). Sites S1 and S2 had the most distinct communities (0.32 wUF distance between sites S1 and S2 at the genus level, 0.11 wUF distance between S3 and S4). Site S1 was overall the most distinct from other sites based on the average wUF distance. S1 microbial community had the lowest alpha diversity among the sites, mainly because of the higher prevalence of algal and cyanobacterial cells (10.7% total combined relative abundance; algal chloroplast DNA and cyanobacterial cells share similar 16S rRNA gene sequence). In contrast, algal + cyanobacterial cells were the least abundant at site S2, consistent with the heaviest shade and the lowest light penetration at this site. In addition to the substantial algal cell presence, all communities were generally dominated by different classes of phylum Proteobacteria, especially Alpha-, Beta-, and Gammaproteo-bacteria (12.9%, 17.8%, and 10.4% on average among sites, respectively, see Figure 2B) [6,11]. Other notable classes included Planctomycetacia (6.7% on average), Gemmatimonadetes (3.6%), and Chitinophagia (2.9%). Combined algal and cyanobacterial counts (denoted as “*Bacillariophyta insertae sedis*” in the annotation database) were also the most abundant at the genus taxonomical level (4.7% on average; 10.5% in the site S1 community). Because of the high prevalence of algal cells in site S1, top 10 genera contributed close to 25% of community counts at this site, higher than the corresponding values for sites S2 (13.9%), S3 (16.9%), and S4 (19.9%, see Figure 2C). Other abundant taxa included Verrucomicrobia subdivision 3 (involved in methane oxidation and carbohydrate degradation in soil) [38], *Nitrospira* (participate in the nitrite-to-nitrate conversions) [39], *Povalibacter*, and Acidobacteria subgroup Gp6 (is positively correlated with the C:N ratio and pH in soil) [40].

In addition to the most abundant genera, we have also examined genera that displayed the widest fluctuations in abundance among sites (defined as taxa with the largest coefficient of variation in relative abundances). Several of them represented algae and Cyanobacteria (Bacillariophyta, taxon GpVIII, *Dulcicalothrix*), all being significantly more prevalent in site S1. A number of aerobic organisms (*Fabibacter*, *Haliscomenobacter*, *Herpetosiphon*, *Hyphomonas*, *Luteimonas*, *Namhaeicola*, and *Parasegetibacter*) were also more abundant at this site, consistent with the higher dissolved oxygen availability in the open stream. Among the above genera, several (*Fabibacter*, GpVIII, *Luteimonas*) are also capable of phosphorus scavenging [41,42,43], consistent with the highest SRP contents at this location.

Finally, for a number of genera, their abundance was inversely associated with the size of the site’s riparian zone, possibly indicating a preference for nutrients from agricultural runoff and human waste. The top five negatively associated genera (as determined by the Spearman correlation coefficient) were *Albidiferax*, *Methylotenera*, *Sulfuritortus* (all from class Betaproteobacteria), *Arcicella* (class Cytophagia), and *Marimicrobium* (Gammaproteobacteria). Among these, *Methylotenera*, which comprise chemo- and methylotrophic members [44], has been previously found at higher abundances in freshwater sediments that received agricultural land runoff compared to control sites not affected by farming [10].

### 3.3. Microbial Composition Is Shaped by the Stream Environment

**Figure 3:**
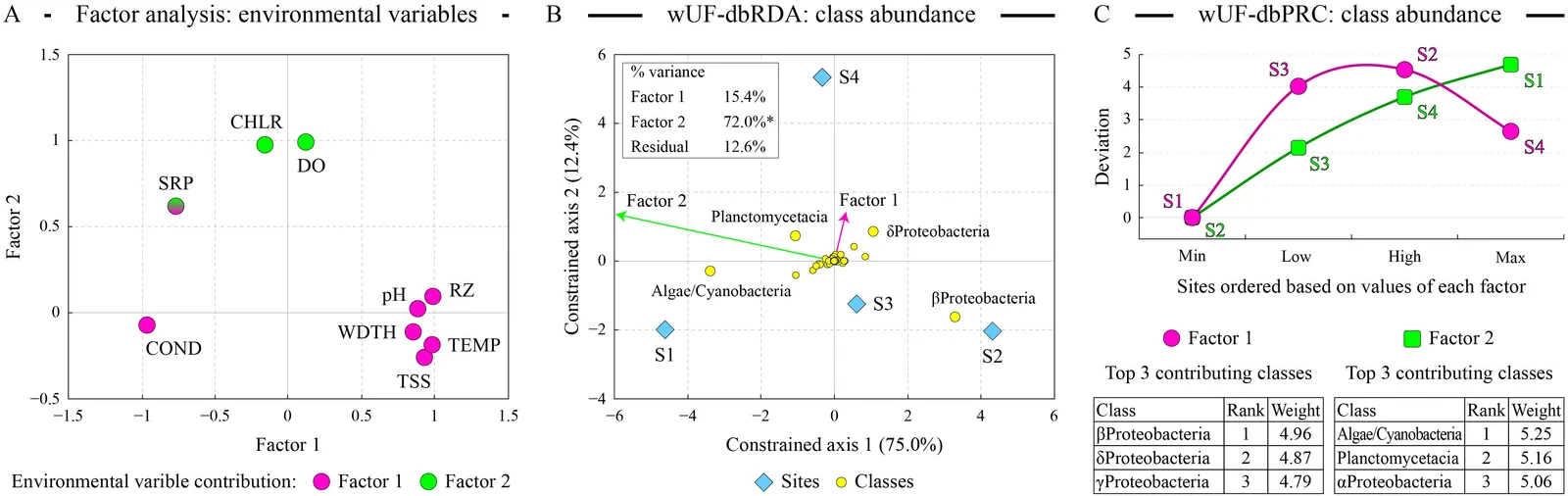
Factor analysis based constrained ordination. (**A**) depicts the contribution of each environmental variable to factors 1 and 2 of the Factor Analysis output. The results of the weighted UniFrac distance-based Redundancy Analysis (wUF-dbRDA) are presented in a triplot in (**B**). Sites are shown as diamonds, factor variables are shown as arrows (with arrow length proportional to the variance explained by that factor), and response variables (microbial classes) as circles. Few notable classes linked to factors and sites are named. The inset displays the distribution of variance between factors and a residual. *: *p* < 0.05.To generate (**C**), the sites were ordered based on the values for each factor, and the microbiota class abundance dataset was subjected to the wUF distance-based principal response curves (dbPRC) analysis. Microbial composition at the site with lowest factor value was set as the baseline and was compared to the microbial composition at every other site. Note that the order of sites differed between factor 1 (sites order S1, S3, S2, S4) and factor 2 (sites order S2, S3, S4, S1, also see (**B**). Larger deviation from zero on the Y axis represents greater shift in the community structure from that of the baseline. The main microbial contributors to the observed shifts in the community composition are shown in the tables.

Factor analysis (FcA) was employed to reduce the dimensionality of our set of measured environmental parameters to two orthogonal factors. The coefficients of environmental variables within each factor are visualized in Figure 3A. Physical and chemical parameters (WDTH, RZ, TEMP, TSS, COND, and pH) contributed primarily to FcA factor 1 scores, whereas CHLR and DO contributed to FcA factor 2. SRP coefficients were comparable between factors 1 and 2, indicating that SRP contributed to both factors similarly. A constrained dbRDA ordination analysis, which used class abundance dataset as the response variable set, FcA factor 1 and 2 coefficients as explanatory variables, and the weighted UniFrac distance as the distance measure, revealed the contributions of each FcA factor to the observed variance in the microbiota structure. As can be seen in Figure 3B, factor 2 contributed significantly (72.0% explained variance, *p* < 0.05) to the microbiota variance, and substantially more than factor 1 (15.4%), indicating that chlorophyll production and the associated amount of dissolved oxygen in the water (both in turn dependent on the amount of sunlight) affected community variance the most. The overall set of environmental variables, represented by FcA factors 1 and 2, accounted for 87.4% of the overall variance of the microbiota composition among the stream sites, suggesting that microbial communities were largely shaped by environmental conditions. The triplot also confirmed the association of Algae + Cyanobacteria cells with site S1 and Betaproteobacteria with site S2 (see Figure 3B).

To provide further support to these insights, sites were ordered according to each factor coefficient, and wUF-dbPRC analysis was run for each factor ordering to reveal the classes that linked sites and environmental factors (Figure 3C). The top three classes with the highest contributing weights are shown in Figure 3C tables. As we observed in other analyses, Algae/Cyanobacteria, Planctomycetacia, and Alphaproteobacteria were linked to chlorophyll *a* and dissolved oxygen-associated factor 2, whereas other classes of Proteobacteria were associated with physical and chemical parameters contributing to FcA factor 1.

### 3.4. Total Amount of Fatty Acids Differs Among Sites

**Figure 4:**
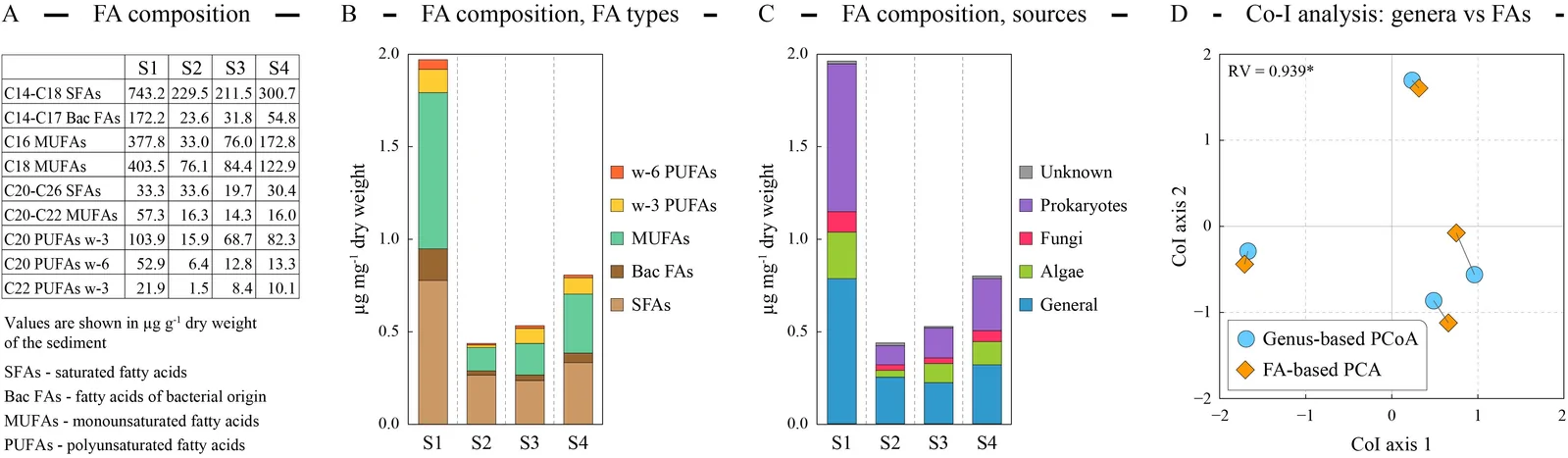
Fatty acid composition of microbial sediments. (**A**) shows the table of fatty acid (FA) abundances. The distributions of cumulative FA abundances among FA types and sources are visualized in (**B**) and (**C**), respectively. Co-Inertia analysis (**D**) shows the congruency of site dispersal in ordination spaces based on (i) the fatty acid and (ii) the class abundance profiles. The distance between each site position on two ordination plots is indicated by a connecting line. Shorter lines represent similar site positioning on each plot. The concordance between the ordinations and its statistical significance were assessed by the RV coefficient and a Monte-Carlo test on the sum of eigenvalues using the *RV.rtest* function in R, respectively. *: *p* < 0.05.

A large number of fatty acid classes were previously measured in the collected river sediments via GC-MS [23] and were analyzed here (Figure 4A,B). The fatty acid (FA) load was by far the highest in site S1 pellet, likely explained by the highest amount of both sunlight and dissolved phosphorus at this site providing energy and nutrients to sustain a higher microbial population density. Though we did not evaluate the composition of fungal communities, the amount of fatty acids classified as of fungal origin was also higher in site S1 (Figure 4C).

Total amount of FAs of algal origin among sites was proportional to the relative abundance of algal + cyanobacterial cells (R_s_ = 1.00). To assess if fatty acid composition was concordant with the community structure among sites, we utilized a Co-Inertia analysis following our previous approach [45]. As shown in Figure 4D, the analysis indicated a strong (RV = 0.939), statistically significant (*p* < 0.05) concordance between site distributions in fatty acid- and microbiota-based ordination spaces.

### 3.5. Analysis of Expressed mRNAs Separates the Communities

**Figure 5:**
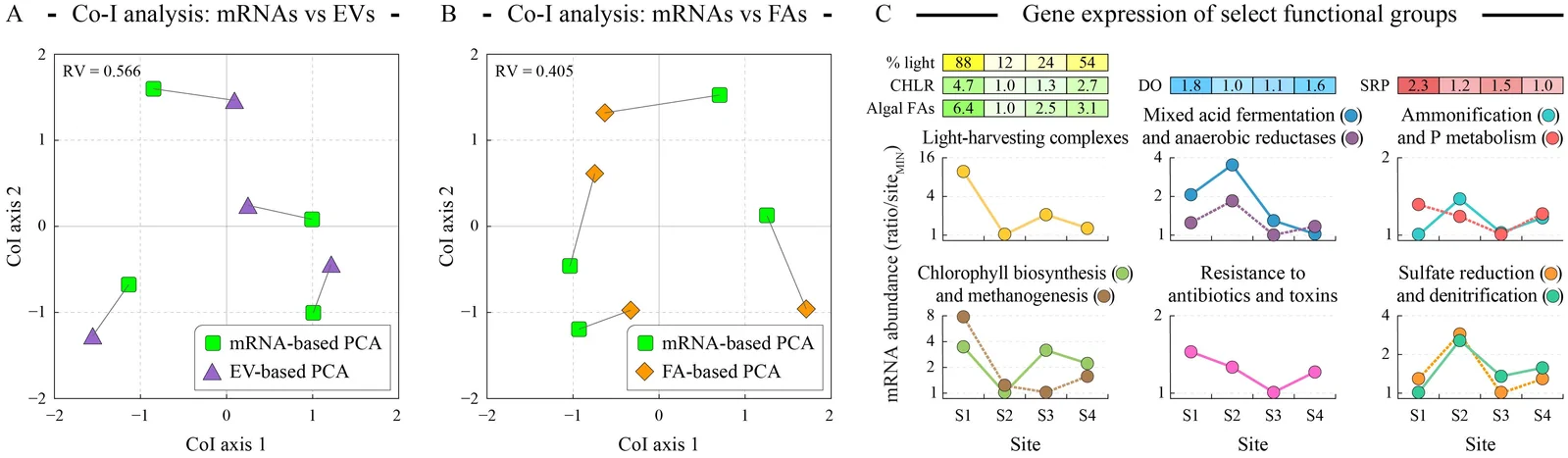
Analysis of microbial gene expression. (**A**,**B**) display the results of Co-Inertia analyses (Co-I) comparing the distribution of sites in ordination spaces based on mRNA gene expression vs. (i) environmental variable differences (EVs, (**A**)) and (ii) fatty acid abundances (FAs, (**B**)). RV coefficient represents the strength of the concordance between the ordinations (minimum: 0, maximum: 1). A selection of specific gene functional groups showing variability in their cumulative expression among the sites is visualized in line graphs of (**C**). All values are displayed as ratios of counts at a particular site to the minimum count value among the four sites. Note that Y axis values are displayed on a log_2_-scale. The associated environmental variables and total algae (as estimated by the fatty acid composition analysis) are shown in boxes with color opacity proportional to the value.

We measured the expression level of abundant microbial mRNAs in all four sites. The distribution of sites in PCA ordination space based on this mRNA expression dataset was similar to the fatty acid- and microbiota-based distributions, though the Co-Inertia analysis comparisons did not achieve the level of statistical significance (Figure 5A,B).

Examination of the expression of functional groups revealed that the overall expression of light-harvesting complexes and genes encoding chlorophyll biosynthesis correlated (R_s_ = 0.80) with the amount of sunlight reaching each site as well as with the total amount of measured chlorophyll and algal fatty acids (Figure 5C left-most panels). We also detected a similar pattern of expression for the methanogenesis functions, which are likely linked to photosynthesis via one-carbon metabolism [46]. The increased expression of methanogenic pathway functions at site S1 might also be linked to the farmland water runoff at this site contributing microbes of animal and fecal origins. While we have not found any evidence of the presence of the typical fecal indicator bacteria such as *Escherichia*, *Salmonella*, *Enterococcus*, and *Streptococcus* at any of the sites, the overall expression of antibiotic resistance genes was elevated at site S1 (Figure 5C, middle bottom panel), consistent with the contamination from surrounding agricultural lands.

The total amount of dissolved oxygen in the water column was inversely associated with mixed acid fermentation and anaerobic respiratory reductases, the functions generally used in anaerobic and microaerophilic conditions (Figure 5C, middle top panel) [47]. The differences among sites were also observed in the expression of microbial functions in the categories of phosphorus metabolism and sulfur and nitrogen reduction and assimilation (Figure 5C, right panels). Phosphorus metabolism genes were expressed the most in the communities of site S1, concordant with the highest abundance of dissolved phosphorus (SRP) at that site. Sulfur and nitrogen reduction pathways as well as ammonification processes were most active in the sediment of site S2, which was the site that received the lowest amount of sunlight.

## 4. Discussion

In this riverbed microbiota and fatty acid analysis, we profiled sediment microbial communities from four distinct sites shown in Figure 1. The sites differed with respect to river width, amount of received sunlight, and the size of the riparian zone. Microbial communities were analyzed for taxonomic composition, functional gene expression, and overall production of fatty acids. All datasets supported similar site-level patterns as can be evidenced by the congruence of site distribution in ordination analysis spaces (See Figure 4D and Figure 5A,B).

The environmental variations among sites gave rise to the differences in microbiota community structure and function, as well as in community fatty acid profiles. For example, site S1 was chosen in the narrow river section fully exposed to sunlight, lacked a riparian zone, and was running through a farmland. Such conditions supported the dominance of algal and cyanobacterial cells in the community, a consistent finding based both on the microbiota analysis and on the fatty acid composition profiling (see Figure 2B,C and Figure 4C). Gene expression analysis provided further evidence by revealing the vastly higher expression of light-harvesting complexes and chlorophyll biosynthesis functions at this site (see Figure 5C). The availability of sunlight at this site, coupled with the high mineral influx based on the soluble phosphorus measurements, generated beneficial conditions for algal/cyanobacterial expansion, which can in turn support the growth of prokaryotic lineages [7]. Algae have been recently shown to play a central role in shaping benthic community structure, likely by producing dissolved organic compounds that become available to other microbes [7]. The previously noted close physical proximity of algae and heterotrophic bacteria in benthic biofilms [5] provides further support to this hypothesis.

Bacterial classes that showed the strongest positive correlations with algal + cyanobacterial abundance included Chitinophagia and Saprospiria (both from phylum Bacteroidota), Chloroflexia, Acidimicrobiia, and Acidobacteria taxon Gp3. In marine environments, members of Bacteroidota were shown to proliferate during algal blooms thanks to their ability to degrade phytoplankton-derived dissolved organic carbon (including algal polysaccharides) [48]. At the same time, the availability of oxygen likely reduced the reliance of communities on sulfate and nitrate reduction [49].

In contrast, site S2 was the most heavily shaded, resulting in low light penetration and reduced dissolved oxygen levels. As a result, algal and cyanobacterial presence was minimal, accompanied by increased numbers of Proteobacteria, lower overall biomass (based on the total amount of measured fatty acids in the sediment, see Figure 4C), and more evenly distributed microbial communities. Anaerobic processes dominated including the expression of mixed acid fermentation enzymes, anaerobic reductases, and functions of ammonification, denitrification, and sulfate reduction (see Figure 5C). We speculate that chemotrophic processes were more prevalent in the site S2 community, which was enriched in members of Betaproteobacteria, a bacterial class comprising microbes capable of denitrification and ammonification in sediments [50]. Two other abundant taxa at this site, Acidobacteria and Verrucomicrobia, were also shown to participate in ammonification and denitrification processes [51,52], and marine Gemmatimonadota can engage in denitrification and sulfate reduction [53]. Among the genera with the highest differential abundance among sites, betaproteobacterial genera *Alicycliphilus*, *Hydrogenophaga*, and *Sulfuricella* (all were the most abundant at site S2) are considered to be strong denitrifiers, and the latter two genera can also participate in sulfur cycling [54,55,56].

The limitations of the current study included only profiling benthic environment in a single season (autumn), the low number of independently profiled river sites (*n* = 4), measuring several environmental parameters in the water column rather than sediment (e.g., dissolved oxygen), and focusing on the prokaryotic microorganisms. While our data are consistent with the hypothesis that phototrophic microorganisms play an important role in benthic community metabolism, further multi-kingdom functional analyses will be required to fully confirm this relationship.

We did not detect any appreciable abundance of typical fecal indicator bacteria at any of our sites, indicating low direct contamination of river waters with farm and human waste at the sampled locations. Nevertheless, the combination of the surrounding land use and the size of the riparian zone was linked to several other community functions including the expression of phosphorus scavenging genes, antibiotic and toxin resistance genes, and methanogenic functions. For example, the concentration of soluble reactive phosphorus was inversely collinear with riparian zone size and, to a lesser degree, was concordant with the expression of the phosphorus scavenging and metabolism functions in the riverbed microbial communities (see Table 1 and Figure 5C). Multiple taxa showed positive or negative associations with riparian zone size across our studied sites as we describe in the results. These observations align well with recent report that demonstrated strong effect of agricultural land use on microbial community diversity, composition, and assembly processes [9].

In summary, both anthropogenic and abiotic factors were linked to riverbed sediment microbial community structure and function. This study shows that environmental conditions such as sunlight, dissolved oxygen, phosphorus availability, riparian zone width, and surrounding agricultural land use can be statistically associated with microbial composition and activity. Since these microbial communities play a critical role in nutrient cycling, organic matter decomposition, and the maintenance of river ecosystem health, understanding their responses to environmental changes is essential. Such knowledge can contribute to more informed management, conservation, and restoration of freshwater ecosystems.

## Figures and Tables

**Table 1:** Measured environmental variables *.

Property	Abbreviation	Units	Site S1	Site S2	Site S3	Site S4
Riparian zone width	RZ	m	0	428	71	1734
River width	WDTH	m	7.0	13.6	5.3	16.6
Light penetration	LIGHT	%	88	12	24	54
Chlorophyll *a* ^†^	CHLR	µg L^−1^	0.66 ± 0.06	0.14 ± 0.08	0.18 ± 0.07	0.38 ± 0.06
Soluble reactive phosphorus ^†^	SRP	mg L^−1^	1.12 ± 0.18	0.56 ± 0.08	0.74 ± 0.03	0.48 ± 0.05
Total suspended solids ^†^	TSS	mg L^−1^	2.52 ± 0.55	4.80 ± 0.52	3.23 ± 0.49	6.22 ± 1.13
Dissolved oxygen	DO	g L^−1^	12.95	7.20	8.10	11.50
pH	pH	pH units	6.82	6.95	7.50	8.20
Conductivity	COND	µS	753	742	729	672
Temperature	TEMP	°C	12.0	12.6	12.5	13.4

* The data are taken from [23]. ^†^ For measurements carried out in the laboratory, the data are shown as arithmetic mean ± standard deviation of technical replicates.

## Data Availability

High-throughput sequencing data are available from the Sequence Read Archive, accession number PRJNA1458852.

## Supplementary Materials

The following supporting information can be downloaded at: https://www.mdpi.com/article/10.3390/microorganisms14102406/s1, Supplementary Table S1: Cumulative table of genus-level cell counts. Supplementary Table S2: Cumulative table of L3-level expressed functions. Supplementary Table S3: Cumulative table of measured fatty acids.
