# Supplementary material for: Light Penetration, Dissolved Oxygen, and Riparian Zone Extent Are Associated with the Structure, Gene Expression, and Lipid Production of Riverbed Microbial Communities
Source: Microorganisms. 2026 Oct 21;14(10):2406. doi: 10.3390/microorganisms14102406 (PMC13707542; doi:10.3390/microorganisms14102406)
Supplement: Supplementary file 1 [file microorganisms-14-02406-s001.zip › Supplementary Table S1. Cumulative table of genus-level cell counts.pdf]

Genus abundance counts	Corresponding class	Site S1	Site S2	Site S3	Site S4
Abyssivirga	Clostridia	0	1	0	0
Achromobacter	Betaproteobacteria	1	1	0	1
Acidibacter	Gammaproteobacteria	65	72	69	71
Acidibrevibacterium	Alphaproteobacteria	0	3	2	2
Acidicaldus	Alphaproteobacteria	0	0	0	1
Acidiferrimicrobium	Acidimicrobiia	0	1	2	0
Acidiferrobacter	Gammaproteobacteria	2	2	2	2
Acidisoma	Alphaproteobacteria	3	2	5	4
Acidisphaera	Alphaproteobacteria	0	2	0	5
Aciditerrimonas	Acidimicrobiia	9	38	36	25
Acidothermus	Actinobacteria	2	0	2	1
Acidovorax	Betaproteobacteria	37	63	33	23
Acinetobacter	Gammaproteobacteria	0	0	1	0
Actinomarinicola	Acidimicrobiia	34	68	52	52
Actinotalea	Actinobacteria	1	0	0	0
Adhaeribacter	Cytophagia	0	4	0	0
Aeromonas	Gammaproteobacteria	0	5	9	5
Aetherobacter	Deltaproteobacteria	2	20	19	16
Afifella	Alphaproteobacteria	0	1	1	2
Afipia	Alphaproteobacteria	5	16	16	11
Agarilytica	Gammaproteobacteria	0	0	2	0
Aggregatilinea	Anaerolineae	58	100	80	118
Aggregicoccus	Deltaproteobacteria	40	32	39	45
Agitococcus	Gammaproteobacteria	1	2	0	2
Agromyces	Actinobacteria	0	2	2	1
Akkermansia	Verrucomicrobiae	1	0	0	1
Albibacterium	Sphingobacteriia	8	4	5	7
Albidiferax	Betaproteobacteria	15	14	12	6
Albidovulum	Alphaproteobacteria	0	1	2	3
Algibacter	Flavobacteriia	0	0	0	1
Algicoccus	Betaproteobacteria	0	1	0	1
Algicola	Gammaproteobacteria	1	1	0	1
Algisphaera	Phycisphaerae	46	43	35	74
Algivirga	Cytophagia	0	0	1	1
Algorimarina	Deltaproteobacteria	2	3	1	1
Algoriphagus	Cytophagia	1	2	3	0
Alicycliphilus	Betaproteobacteria	2	24	4	5
Aliidongia	Alphaproteobacteria	21	22	15	26
Aliterella	Cyanobacteria	0	1	0	0
Alkalinema	Cyanobacteria	9	0	0	0
Alkalitalea	Bacteroidia	1	1	2	3
Alkanindiges	Gammaproteobacteria	0	1	1	2
Allosphingosinicella	Alphaproteobacteria	1	5	1	3
Allostreptomyces	Actinobacteria	1	0	1	0
Alsobacter	Alphaproteobacteria	8	8	9	8
Altererythrobacter	Alphaproteobacteria	23	62	61	43
Alterococcus	Opitutae	6	9	16	7
Amaricoccus	Alphaproteobacteria	2	1	0	2
Aminipila	Clostridia	1	0	1	0

Genus abundance counts	Corresponding class	Site S1	Site S2	Site S3	Site S4
Aminivibrio	Synergistia	0	3	3	11
Aminobacter	Alphaproteobacteria	2	1	1	0
Amorphus	Alphaproteobacteria	5	5	4	6
Amphiplicatus	Alphaproteobacteria	5	5	7	16
Ampullimonas	Betaproteobacteria	15	46	31	28
Anaerobacterium	Clostridia	1	1	1	0
Anaerohalosphaera	Phycisphaerae	28	30	29	29
Anaerolinea	Anaerolineae	11	6	6	8
Anaeromyxobacter	Deltaproteobacteria	74	45	39	66
Anaerorhabdus	Bacteroidia	0	1	0	0
Ancylomarina	Bacteroidia	0	1	3	0
Anderseniella	Alphaproteobacteria	56	42	84	126
Angustibacter	Actinobacteria	1	0	0	0
Annwoodia	Betaproteobacteria	19	26	21	29
Anseongella	Sphingobacteriia	7	2	6	11
Apibacter	Flavobacteriia	1	1	3	2
Aquabacter	Alphaproteobacteria	5	1	4	3
Aquabacterium	Betaproteobacteria	91	144	89	79
Aquicella	Gammaproteobacteria	1	0	1	3
Aquidulcibacter	Alphaproteobacteria	61	23	42	55
Aquiflexum	Cytophagia	1	0	0	1
Aquihabitans	Acidimicrobiia	24	17	23	24
Aquimonas	Gammaproteobacteria	20	19	19	19
Aquincola	Betaproteobacteria	54	88	56	39
Aquirufa	Cytophagia	0	2	1	3
Aquisalinus	Alphaproteobacteria	2	3	1	0
Aquisediminimonas	Alphaproteobacteria	14	11	25	11
Aquisphaera	Planctomycetacia	7	7	6	8
Arboricoccus	Alphaproteobacteria	2	5	3	3
Archangium	Deltaproteobacteria	0	1	0	1
Arcicella	Cytophagia	20	13	15	5
Ardenticatena	Ardenticatenia	2	2	2	4
Arenicella	Gammaproteobacteria	0	0	0	1
Arenimicrobium	Blastocatellia	0	1	3	2
Arenimonas	Gammaproteobacteria	61	39	49	44
Aridibacter	Blastocatellia	16	4	10	12
Armatimonadetesgp2	Armatimonadia	0	2	2	0
Armatimonadetesgp4	Armatimonadia	0	2	0	1
Armatimonadetesgp5	Armatimonadia	13	16	15	15
ArmatimonasArmatimonadetesgp1	Armatimonadia	6	5	3	6
Aromatoleum	Betaproteobacteria	6	15	15	4
Arsenicitalea	Alphaproteobacteria	0	1	0	1
Arsenophonus	Gammaproteobacteria	0	0	1	0
Arvibacter	Chitinophagia	1	0	1	0
Asprobacter	Alphaproteobacteria	114	65	89	76
Asticcacaulis	Alphaproteobacteria	0	1	0	0
Aurantimicrobium	Actinobacteria	1	16	19	8
Aureimonas	Alphaproteobacteria	0	1	0	0
Aureispira	Saprospiria	1	1	2	3

Genus abundance counts	Corresponding class	Site S1	Site S2	Site S3	Site S4
Azoarcus	Betaproteobacteria	0	3	0	0
Azohydromonas	Betaproteobacteria	25	36	22	31
Azomonas	Gammaproteobacteria	0	0	3	2
Azonexus	Betaproteobacteria	5	10	9	8
Azospira	Betaproteobacteria	8	31	23	16
Azovibrio	Betaproteobacteria	6	12	6	7
Bacillariophyta	Chloroplast	1849	119	577	762
Bacillus	Bacilli	2	2	1	2
Bacterioplanes	Gammaproteobacteria	0	1	0	1
Bacteriovorax	Oligoflexia	4	3	4	5
Bacteroides	Bacteroidia	0	0	0	1
Baekduia	Thermoleophilia	5	12	10	15
Basilea	Betaproteobacteria	0	5	2	1
Bauldia	Alphaproteobacteria	3	14	13	15
Bdellovibrio	Deltaproteobacteria	26	54	52	67
Bellilinea	Anaerolineae	42	65	73	114
Blastocatella	Blastocatellia	13	9	7	9
Blastochloris	Alphaproteobacteria	0	1	0	0
Blastomonas	Alphaproteobacteria	2	6	13	13
Blastopirellula	Planctomycetacia	23	17	12	21
Bordetella	Betaproteobacteria	0	1	1	2
Bosea	Alphaproteobacteria	0	1	2	1
Brachymonas	Betaproteobacteria	0	1	0	0
Bradyrhizobium	Alphaproteobacteria	9	18	21	4
BRC1generaincertaesedis	BRC1	1	0	4	1
Brevifollis	Verrucomicrobiae	32	10	11	12
Brevinema	Spirochaetia	0	0	2	0
Brevitalea	Blastocatellia	1	3	2	2
Brevundimonas	Alphaproteobacteria	3	1	0	1
Breznakibacter	Bacteroidia	9	10	11	11
Brumimicrobium	Flavobacteriia	1	1	1	1
Bryobacter	Acidobacteria_Gp3	2	0	0	1
Bryocella	Acidobacteria_Gp1	1	1	0	1
Butyratibacter	Alphaproteobacteria	0	0	1	0
Byssovorax	Deltaproteobacteria	15	25	21	19
Bythopirellula	Planctomycetacia	26	21	20	14
Caenibius	Alphaproteobacteria	2	3	0	1
Caenimonas	Betaproteobacteria	11	19	11	12
Caldilinea	Caldilineae	5	3	3	0
Caldimonas	Betaproteobacteria	4	7	7	4
Caldovatus	Alphaproteobacteria	0	0	0	1
Calorithrix	Deferribacteres	12	25	31	34
CandidatusAnammoxoglobus	Candidatus Anammoxoglobus	10	5	7	10
CandidatusBrocadia	Candidatus Brocadia	24	13	34	31
CandidatusCarsonella	Candidatus Carsonella	8	1	11	13
CandidatusHydrogenedens	Candidatus Hydrogenedens	12	39	56	45
CandidatusIainarchaeum	Candidatus Iainarchaeum	0	0	2	0
CandidatusKoribacter	Candidatus Koribacter	2	4	2	3
CandidatusKuenenia	Candidatus Kuenenia	3	6	11	16

Genus abundance counts	Corresponding class	Site S1	Site S2	Site S3	Site S4
CandidatusSolibacter	Candidatus Solibacter	22	9	13	8
Casimicrobium	Betaproteobacteria	18	27	23	17
Catabacter	Clostridia	0	1	1	0
Caulobacter	Alphaproteobacteria	0	3	4	3
Cavicella	Gammaproteobacteria	2	2	1	2
Cellvibrio	Gammaproteobacteria	13	25	27	17
Cephalothrix	Cyanobacteria	1	0	1	1
Cephaloticoccus	Opitutae	2	1	40	6
Chakrabartia	Alphaproteobacteria	0	1	0	2
Chelativorans	Alphaproteobacteria	5	3	1	1
Chiayiivirga	Gammaproteobacteria	5	3	1	5
Chitinibacter	Betaproteobacteria	1	2	1	3
Chitinimonas	Betaproteobacteria	20	9	14	6
Chitinispirillum	Chitinispirillia	1	3	3	4
Chitinivorax	Betaproteobacteria	0	2	0	0
Chitinophaga	Chitinophagia	1	1	1	2
Chlorophyta	Chloroplast	16	0	1	0
Chondromyces	Deltaproteobacteria	3	2	1	4
Chromatocurvus	Gammaproteobacteria	16	40	39	38
Chryseolinea	Cytophagia	236	145	202	145
Chryseotalea	Cytophagia	36	22	30	38
ChthonomonasArmatimonadetesgp3	Clostridia	1	0	0	2
Clostridiumsensustricto	Clostridia	3	7	1	3
ClostridiumXlVa	Clostridia	0	1	0	0
Cnuella	Chitinophagia	1	0	0	0
Cohaesibacter	Alphaproteobacteria	2	1	1	0
Collimonas	Betaproteobacteria	0	0	1	3
Comamonas	Betaproteobacteria	4	11	11	5
Conexibacter	Thermoleophilia	5	5	3	8
Coraliomargarita	Opitutae	0	0	0	1
Corallococcus	Deltaproteobacteria	0	2	0	1
Coralloluteibacterium	Gammaproteobacteria	6	9	12	7
Corynebacterium	Actinobacteria	0	0	1	0
Coxiella	Gammaproteobacteria	0	2	1	7
Crinalium	Cyanobacteria	14	0	2	0
Croceicoccus	Alphaproteobacteria	0	2	1	0
Crocinitomix	Flavobacteriia	17	15	11	18
Cronbergia	Cyanobacteria	13	1	2	2
Cryobacterium	Actinobacteria	1	1	3	1
Cryptanaerobacter	Clostridia	0	1	0	0
Cryptomonadaceae	Chloroplast	0	1	2	10
Cucumibacter	Alphaproteobacteria	1	0	0	0
Curvibacter	Betaproteobacteria	60	116	70	41
Cypionkella	Alphaproteobacteria	17	16	19	17
Cystobacter	Deltaproteobacteria	1	3	2	1
Cytobacillus	Bacilli	0	1	1	0
Cytophaga	Cytophagia	3	2	2	5
Daeguia	Alphaproteobacteria	1	0	0	0
Dasania	Gammaproteobacteria	1	4	1	2

Genus abundance counts	Corresponding class	Site S1	Site S2	Site S3	Site S4
Dechloromonas	Betaproteobacteria	83	131	59	28
Deefgea	Betaproteobacteria	0	2	5	1
Deferrisoma	Deltaproteobacteria	4	5	7	8
Defluviicoccus	Alphaproteobacteria	0	3	5	1
Defluviimonas	Alphaproteobacteria	5	8	7	5
Dehalogenimonas	Dehalococcoidia	0	0	1	1
Deinobacterium	Deinococci	1	0	2	0
Delftia	Betaproteobacteria	5	15	10	4
Demequina	Actinobacteria	0	0	1	1
Denitratisoma	Betaproteobacteria	11	23	14	16
Derxia	Betaproteobacteria	0	2	3	6
Desertibacter	Alphaproteobacteria	3	2	3	6
Desertimonas	Acidimicrobiia	173	50	58	75
Desulfacinum	Deltaproteobacteria	2	0	0	1
Desulfatiglans	Deltaproteobacteria	4	10	8	10
Desulfatirhabdium	Deltaproteobacteria	11	6	5	11
Desulfatitalea	Deltaproteobacteria	7	11	8	21
Desulfobacca	Deltaproteobacteria	4	0	5	5
Desulfobacula	Deltaproteobacteria	0	1	1	1
Desulfobulbus	Deltaproteobacteria	16	16	11	6
Desulfocapsa	Deltaproteobacteria	11	11	5	6
Desulfocarbo	Deltaproteobacteria	0	0	1	0
Desulfoconvexum	Deltaproteobacteria	1	1	0	0
Desulfofaba	Deltaproteobacteria	0	0	0	1
Desulfofustis	Deltaproteobacteria	1	0	1	1
Desulfoglaeba	Deltaproteobacteria	6	9	4	9
Desulfomicrobium	Deltaproteobacteria	1	6	1	2
Desulfomonile	Deltaproteobacteria	6	4	3	4
Desulfonatronobacter	Deltaproteobacteria	1	0	2	2
Desulfopila	Deltaproteobacteria	1	4	1	2
Desulfoprunum	Deltaproteobacteria	3	3	1	1
Desulforhabdus	Deltaproteobacteria	6	3	4	10
Desulforhopalus	Deltaproteobacteria	17	10	9	9
Desulfosarcina	Deltaproteobacteria	0	1	0	0
Desulfospira	Deltaproteobacteria	1	0	3	1
Desulfothermobacter	Clostridia	1	10	7	4
Desulfovermiculus	Deltaproteobacteria	0	1	1	3
Desulfovibrio	Deltaproteobacteria	1	2	0	0
Desulfovirga	Deltaproteobacteria	10	12	16	23
Desulfurivibrio	Deltaproteobacteria	0	2	0	0
Desulfuromonas	Deltaproteobacteria	14	101	68	47
Dethiosulfatarculus	Deltaproteobacteria	6	10	19	24
Devosia	Alphaproteobacteria	2	4	3	6
Diaphorobacter	Betaproteobacteria	20	30	9	6
Dinghuibacter	Chitinophagia	0	0	1	0
Dissulfuribacter	Deltaproteobacteria	0	1	1	1
Dissulfurimicrobium	Deltaproteobacteria	18	13	21	26
Dissulfurirhabdus	Deltaproteobacteria	8	17	16	13
Dokdonella	Gammaproteobacteria	2	3	3	4

Genus abundance counts	Corresponding class	Site S1	Site S2	Site S3	Site S4
Dongia	Alphaproteobacteria	5	17	12	17
Duganella	Betaproteobacteria	0	4	5	5
Dulcicalothrix	Cyanobacteria	43	6	9	4
Duodenibacillus	Betaproteobacteria	1	1	1	1
Dysgonomonas	Bacteroidia	0	1	0	1
Edaphobaculum	Chitinophagia	4	2	1	5
Eionea	Gammaproteobacteria	0	1	3	3
Elioraea	Alphaproteobacteria	0	0	1	1
Elkelangia	Alphaproteobacteria	2	1	4	2
Elstera	Alphaproteobacteria	1	5	4	6
Elusimicrobium	Elusimicrobia	4	1	4	0
Embleya	Actinobacteria	2	1	2	1
Emticicia	Cytophagia	1	1	1	3
Endomicrobium	Endomicrobia	12	6	1	6
Endothiovibrio	Gammaproteobacteria	2	0	5	4
Enhygromyxa	Deltaproteobacteria	2	4	6	11
Enteroscipio	Coriobacteriia	0	0	0	1
Ercella	Clostridia	0	0	0	2
Ereboglobus	Opitutae	9	4	0	2
Erythrobacter	Alphaproteobacteria	2	1	8	2
Erythromicrobium	Alphaproteobacteria	1	0	0	0
EscherichiaShigella	Gammaproteobacteria	0	0	1	1
Euryhalocaulis	Alphaproteobacteria	11	8	13	11
Euzebya	Nitriliruptoria	1	1	2	1
Fabibacter	Cytophagia	16	4	4	3
Faecalibacter	Flavobacteriia	2	0	1	2
Falsirhodobacter	Alphaproteobacteria	2	0	1	0
Ferribacterium	Betaproteobacteria	2	6	2	1
Ferriphaselus	Betaproteobacteria	0	0	1	1
Ferritrophicum	Betaproteobacteria	8	36	32	36
Ferrovibrio	Alphaproteobacteria	1	2	0	0
Ferruginibacter	Chitinophagia	166	57	119	50
Ferruginivarius	Alphaproteobacteria	1	4	5	9
Fictibacillus	Bacilli	0	0	0	1
Filomicrobium	Alphaproteobacteria	17	7	14	32
Fimbriiglobus	Planctomycetacia	40	24	19	33
Flaviaesturariibacter	Chitinophagia	1	0	1	1
Flavihumibacter	Chitinophagia	36	13	23	1
Flavilitoribacter	Saprospiria	6	9	11	8
Flavisolibacter	Chitinophagia	1	1	0	0
Flavitalea	Chitinophagia	90	29	35	34
Flavobacterium	Flavobacteriia	41	36	53	63
Flectobacillus	Cytophagia	0	2	2	1
Flexilinea	Anaerolineae	0	1	2	1
Fluviicoccus	Gammaproteobacteria	1	0	1	1
Fluviicola	Flavobacteriia	10	8	12	17
Fluviispira	Oligoflexia	2	3	2	2
Foliisarcina	Cyanobacteria	1	0	0	0
Formivibrio	Betaproteobacteria	1	8	3	1

Genus abundance counts	Corresponding class	Site S1	Site S2	Site S3	Site S4
Formosimonas	Betaproteobacteria	0	1	1	0
Friedmanniella	Actinobacteria	0	2	0	0
Fulvimonas	Gammaproteobacteria	1	5	6	1
Fulvivirga	Cytophagia	1	1	3	1
Fuscovulum	Alphaproteobacteria	1	0	2	2
Fusibacter	Clostridia	0	2	0	0
Gaiella	Thermoleophilia	44	136	125	87
Galactobacter	Actinobacteria	0	1	0	1
Galbitalea	Actinobacteria	0	0	1	0
Gallionella	Betaproteobacteria	0	1	0	3
Geminicoccus	Alphaproteobacteria	0	1	1	0
Geminisphaera	Opitutae	1	0	0	0
Gemmata	Planctomycetacia	10	6	9	12
Gemmatimonas	Gemmatimonadetes	293	211	253	226
Gemmatirosa	Gemmatimonadetes	82	183	203	201
Gemmobacter	Alphaproteobacteria	71	50	45	37
Geobacter	Deltaproteobacteria	49	112	62	54
Geofilum	Bacteroidia	0	1	2	1
Geomonas	Deltaproteobacteria	2	3	3	4
Geopsychrobacter	Deltaproteobacteria	1	11	10	6
Georgfuchsia	Betaproteobacteria	61	199	139	116
Geothermobacter	Deltaproteobacteria	2	1	3	2
Geothrix	Holophagae	6	17	25	13
Gimesia	Planctomycetacia	35	17	19	31
Gp10	Acidobacteria_Gp10	10	20	12	17
Gp13	Acidobacteria_Gp13	1	1	1	1
Gp16	Acidobacteria_Gp16	18	40	43	37
Gp17	Acidobacteria_Gp17	53	69	72	90
Gp18	Acidobacteria_Gp18	6	15	15	15
Gp2	Acidobacteria_Gp2	0	2	4	4
Gp22	Acidobacteria_Gp22	16	31	26	32
Gp23	Acidobacteria_Gp23	0	0	1	0
Gp25	Acidobacteria_Gp25	0	2	0	0
Gp3	Acidobacteria_Gp3	60	21	22	36
Gp4	Acidobacteria_Gp4	2	13	9	9
Gp6	Acidobacteria_Gp6	271	342	256	283
Gp7	Acidobacteria_Gp7	22	48	61	41
Gp9	Acidobacteria_Gp9	0	2	0	0
GpI	Cyanobacteria	18	1	0	0
GpIIa	Cyanobacteria	2	0	0	0
GpIV	Cyanobacteria	3	0	0	0
GpV	Cyanobacteria	1	0	0	1
GpVI	Cyanobacteria	7	0	2	9
GpVIII	Cyanobacteria	19	1	1	0
GpXIII	Cyanobacteria	1	0	1	1
Gracilibacter	Clostridia	1	1	1	0
Gynuella	Gammaproteobacteria	0	0	4	3
Haematobacter	Alphaproteobacteria	1	0	1	0
Halarcobacter	Campylobacteria	0	3	0	0

**Genus abundance counts**	**Corresponding class**	Site S1	Site S2	Site S3	Site S4
Haliangium	Deltaproteobacteria	13	10	17	21
Haliea	Gammaproteobacteria	0	4	11	6
Halioglobus	Gammaproteobacteria	13	6	15	12
Haliscomenobacter	Saprospiria	17	4	2	7
Halobacteriovorax	Oligoflexia	2	7	10	8
Halochromatium	Gammaproteobacteria	2	0	1	1
Halodurantibacterium	Alphaproteobacteria	4	0	0	0
Halofilum	Gammaproteobacteria	5	2	1	1
Halospirulina	Cyanobacteria	8	0	0	0
Halovulum	Alphaproteobacteria	2	3	5	1
Haoranjiania	Chitinophagia	1	1	2	0
Hartmannibacter	Alphaproteobacteria	3	1	2	0
Heliimonas	Chitinophagia	13	4	6	8
Herminiimonas	Betaproteobacteria	3	1	4	2
Herpetosiphon	Chloroflexia	23	0	0	1
Hoeflea	Alphaproteobacteria	11	5	1	1
Holophaga	Holophagae	0	1	0	1
Hyalangium	Deltaproteobacteria	3	1	0	0
Hydrogenispora	Clostridia	0	3	1	1
Hydrogenobaculum	Aquificae	0	0	2	1
Hydrogenophaga	Betaproteobacteria	17	87	20	10
Hyphococcus	Alphaproteobacteria	8	4	4	9
Hyphomicrobium	Alphaproteobacteria	168	167	234	290
Hyphomonas	Alphaproteobacteria	24	3	5	1
Hypnocyclicus	Fusobacteriia	0	3	0	0
Iamia	Acidimicrobiia	4	3	6	9
Ichthyobacterium	Flavobacteriia	0	1	0	0
Ideonella	Betaproteobacteria	67	83	80	76
Ignavibacterium	Ignavibacteria	81	32	49	72
Ilumatobacter	Acidimicrobiia	108	23	36	43
Ilyomonas	Chitinophagia	2	1	2	0
Immundisolibacter	Gammaproteobacteria	0	2	3	4
Imperialibacter	Cytophagia	0	2	1	1
Indibacter	Cytophagia	0	0	1	1
Inhella	Betaproteobacteria	33	22	14	17
Inquilinus	Alphaproteobacteria	0	0	0	1
Iphinoe	Cyanobacteria	3	0	0	0
Jahnella	Deltaproteobacteria	17	27	28	25
Janibacter	Actinobacteria	0	1	0	0
Jannaschia	Alphaproteobacteria	1	0	0	0
Janthinobacterium	Betaproteobacteria	1	0	0	0
Jiangella	Actinobacteria	0	3	2	1
Kallotenue	Chloroflexia	0	0	0	2
Ketobacter	Gammaproteobacteria	0	2	2	4
Kineobactrum	Gammaproteobacteria	4	7	7	7
Kinneretia	Betaproteobacteria	2	0	0	2
Kiritimatiella	Kiritimatiellae	8	6	5	8
Kofleria	Deltaproteobacteria	46	89	102	117
Kribbella	Actinobacteria	0	2	1	0

Genus abundance counts	Corresponding class	Site S1	Site S2	Site S3	Site S4
Labilithrix	Deltaproteobacteria	15	19	17	32
Lachnotalea	Clostridia	0	1	0	0
Lacibacter	Chitinophagia	98	21	54	9
Lacibacterium	Alphaproteobacteria	1	5	7	10
Lacihabitans	Cytophagia	3	7	18	14
Lacipirellula	Planctomycetacia	404	161	203	235
Lacisediminimonas	Betaproteobacteria	0	9	5	3
Lactococcus	Bacilli	0	0	1	0
Lacunisphaera	Opitutae	8	6	6	6
Laribacter	Betaproteobacteria	0	2	0	0
Latescibacteriageneraincertaesedis	Latescibacteria	31	72	52	67
Lautropia	Betaproteobacteria	1	1	0	0
Legionella	Gammaproteobacteria	1	2	1	0
Leifsonia	Actinobacteria	0	0	2	1
Leminorella	Gammaproteobacteria	0	0	1	1
Lentimicrobium	Bacteroidia	32	30	38	60
Leptolinea	Anaerolineae	5	13	10	7
Leptospirillum	Nitrospira	0	0	1	1
Leptothrix	Betaproteobacteria	58	38	15	6
Leucobacter	Actinobacteria	0	0	1	0
Levilinea	Anaerolineae	0	4	1	2
Limibacillus	Alphaproteobacteria	1	2	3	2
Limisphaera	Subdivision3	10	3	5	3
Limnobacter	Betaproteobacteria	1	2	3	0
Limnoglobus	Planctomycetacia	16	5	6	20
Limnohabitans	Betaproteobacteria	97	109	75	31
Lishizhenia	Flavobacteriia	1	1	2	1
Litoribacillus	Gammaproteobacteria	1	0	1	0
Litorilinea	Caldilineae	53	69	77	83
Longibacter	Rhodothermia	0	0	1	1
Longilinea	Anaerolineae	26	31	30	32
Longimicrobium	Longimicrobia	2	12	13	11
Longivirga	Actinobacteria	9	7	12	10
Loriellopsis	Cyanobacteria	0	1	0	0
Luedemannella	Actinobacteria	0	2	0	0
Luminiphilus	Gammaproteobacteria	3	2	5	5
Luteibacter	Gammaproteobacteria	3	0	1	0
Luteimonas	Gammaproteobacteria	55	22	13	4
Luteitalea	Vicinamibacteria	7	17	9	9
Luteolibacter	Verrucomicrobiae	83	25	111	81
Luteovulum	Alphaproteobacteria	3	5	7	3
Lysinibacillus	Bacilli	0	2	1	1
Lysobacter	Gammaproteobacteria	22	19	15	5
Lyticum	Alphaproteobacteria	0	0	0	2
Magnetococcus	Alphaproteobacteria	0	0	2	0
Magnetospirillum	Alphaproteobacteria	0	1	0	0
Mailhella	Deltaproteobacteria	3	1	2	2
Malaciobacter	Campylobacteria	0	3	0	0
Malikia	Betaproteobacteria	0	6	0	0

Genus abundance counts	Corresponding class	Site S1	Site S2	Site S3	Site S4
Malonomonas	Deltaproteobacteria	2	4	3	1
Mameliella	Alphaproteobacteria	2	1	2	0
Mangrovibacterium	Bacteroidia	1	3	4	2
Mangroviflexus	Bacteroidia	2	0	3	1
Mangrovitalea	Gammaproteobacteria	2	4	1	6
Maribellus	Bacteroidia	16	13	30	17
Marimicrobium	Gammaproteobacteria	75	69	77	48
Marinibactrum	Gammaproteobacteria	0	1	0	1
Marinibaculum	Alphaproteobacteria	0	0	4	1
Mariniblastus	Planctomycetacia	35	27	19	58
Mariniphaga	Bacteroidia	1	2	1	3
Marinoscillum	Cytophagia	0	0	1	1
Marispirochaeta	Spirochaetia	2	6	7	5
Marmoricola	Actinobacteria	1	6	3	1
Massilia	Betaproteobacteria	0	2	1	1
Meiothermus	Deinococci	2	4	3	6
Melaminivora	Betaproteobacteria	0	2	3	2
Melioribacter	Ignavibacteria	124	61	94	99
Melittangium	Deltaproteobacteria	3	7	8	17
Membranicola	Saprospiria	1	1	0	0
Meniscus	Bacteroidia	0	3	1	2
Mesobacillus	Bacilli	1	1	0	0
Mesorhizobium	Alphaproteobacteria	2	11	4	9
Methanomassiliicoccus	Thermoplasmata	0	11	0	0
Methanosarcina	Methanomicrobia	0	0	1	0
Methanothrix	Methanomicrobia	9	0	2	0
Methylibium	Betaproteobacteria	8	4	3	1
Methylobacillus	Betaproteobacteria	0	2	1	1
Methylobacter	Gammaproteobacteria	16	19	15	15
Methylobacterium	Alphaproteobacteria	0	0	0	1
Methylocapsa	Alphaproteobacteria	1	2	4	1
Methyloceanibacter	Alphaproteobacteria	5	43	30	19
Methylocella	Alphaproteobacteria	1	2	5	3
Methylococcus	Gammaproteobacteria	0	1	0	0
Methylocystis	Alphaproteobacteria	4	4	4	5
Methyloferula	Alphaproteobacteria	0	1	3	1
Methyloglobulus	Gammaproteobacteria	6	15	27	9
Methylohalomonas	Gammaproteobacteria	2	1	2	7
Methyloligella	Alphaproteobacteria	1	10	6	11
Methylomagnum	Gammaproteobacteria	1	0	0	2
Methylomarinum	Gammaproteobacteria	4	1	2	2
Methylomicrobium	Gammaproteobacteria	5	7	10	3
Methylomonas	Gammaproteobacteria	2	3	4	4
Methylophilus	Betaproteobacteria	3	4	1	2
Methylosarcina	Gammaproteobacteria	1	2	1	2
Methylosoma	Gammaproteobacteria	0	2	2	2
Methylotenera	Betaproteobacteria	132	132	117	38
Methyloterrigena	Alphaproteobacteria	1	0	1	1
Methyloversatilis	Betaproteobacteria	7	49	27	15

Genus abundance counts	Corresponding class	Site S1	Site S2	Site S3	Site S4
Methylovirgula	Alphaproteobacteria	5	1	1	7
Methylovorus	Betaproteobacteria	1	3	2	1
Methylovulum	Gammaproteobacteria	30	90	196	20
Microbacterium	Actinobacteria	0	1	1	0
Microbaculum	Alphaproteobacteria	2	2	1	2
Microcella	Actinobacteria	0	3	5	1
Microgenomatesgeneraincertaesedis	Microgenomates	0	1	1	0
Micropepsis	Alphaproteobacteria	4	5	6	4
Microvirga	Alphaproteobacteria	0	3	2	2
Minicystis	Deltaproteobacteria	1	12	7	15
Mitsuaria	Betaproteobacteria	0	1	2	0
Mizugakiibacter	Gammaproteobacteria	0	1	1	0
Mongoliimonas	Alphaproteobacteria	0	1	0	1
Motiliproteus	Gammaproteobacteria	0	1	1	2
Mycetohabitans	Betaproteobacteria	5	13	6	5
Mycobacterium	Actinobacteria	0	21	13	11
Nafulsella	Cytophagia	3	0	0	0
Nakamurella	Actinobacteria	0	1	1	0
Namhaeicola	Flavobacteriia	13	1	4	4
Nemorincola	Chitinophagia	4	1	1	1
Neorickettsia	Alphaproteobacteria	1	0	0	0
Niabella	Chitinophagia	0	0	0	1
Niastella	Chitinophagia	0	0	1	0
Nibribacter	Cytophagia	0	0	0	1
Nitratireductor	Alphaproteobacteria	243	98	126	60
Nitrobacter	Alphaproteobacteria	0	5	2	1
Nitrolancea	Thermomicrobia	12	7	7	12
Nitrosomonas	Betaproteobacteria	9	7	2	2
Nitrosopumilus	Nitrosopumilales	8	3	9	17
Nitrososphaera	Nitrososphaerales	4	26	16	18
Nitrosospira	Betaproteobacteria	25	85	57	49
Nitrospira	Nitrospira	347	402	317	447
Niveibacterium	Betaproteobacteria	0	0	2	0
Niveitalea	Chitinophagia	2	0	1	0
Nocardioides	Actinobacteria	25	20	21	24
Noviherbaspirillum	Betaproteobacteria	1	1	2	1
Novimethylophilus	Betaproteobacteria	0	6	2	0
Novosphingobium	Alphaproteobacteria	106	178	210	155
Oblitimonas	Gammaproteobacteria	1	1	1	2
Occallatibacter	Acidobacteria_Gp1	0	1	0	0
Occidentia	Alphaproteobacteria	1	1	6	2
Oceanibacterium	Alphaproteobacteria	0	0	2	4
Oceanicella	Alphaproteobacteria	0	1	2	1
Oceanicoccus	Gammaproteobacteria	0	2	4	10
Oharaeibacter	Alphaproteobacteria	0	0	1	0
Ohtaekwangia	Cytophagia	6	8	12	6
Oleiagrimonas	Gammaproteobacteria	0	0	2	1
Oleiharenicola	Opitutae	36	55	72	93
Oleiphilus	Gammaproteobacteria	0	3	6	9

**Genus abundance counts**	**Corresponding class**	Site S1	Site S2	Site S3	Site S4
Oligoflexus	Oligoflexia	2	2	3	3
Oligosphaera	Oligosphaeria	2	1	3	3
Oligotropha	Alphaproteobacteria	1	9	10	3
Olsenella	Coriobacteriia	0	0	0	1
Omnitrophicageneraincertaesedis	Omnitrophica	1	0	2	0
Opitutus	Opitutae	46	48	70	63
Ornatilinea	Anaerolineae	15	48	37	29
Ornithinibacter	Actinobacteria	0	1	1	2
Oryzisolibacter	Betaproteobacteria	1	0	1	0
Oryzomicrobium	Betaproteobacteria	0	1	0	0
Ottowia	Betaproteobacteria	5	12	2	4
Oxalicibacterium	Betaproteobacteria	10	7	9	7
Oxalobacter	Betaproteobacteria	1	4	1	0
PacearchaeotaIncertaeSedisAR13	Pacearchaeota	2	1	6	2
Paenirhodobacter	Alphaproteobacteria	0	2	2	1
Paludibacter	Bacteroidia	0	1	0	1
Paludibacterium	Betaproteobacteria	2	3	4	4
Paludibaculum	Acidobacteria_Gp3	17	9	2	3
Paludisphaera	Planctomycetacia	7	4	5	13
Panacagrimonas	Gammaproteobacteria	0	1	0	1
Panacibacter	Chitinophagia	3	1	0	0
Pandoraea	Betaproteobacteria	0	0	1	1
Pantanalinema	Cyanobacteria	5	0	1	0
Papillibacter	Clostridia	0	1	0	0
Parablastomonas	Alphaproteobacteria	3	2	4	3
Paraburkholderia	Betaproteobacteria	1	1	2	1
Paraclostridium	Clostridia	0	0	0	1
Paracoccus	Alphaproteobacteria	59	33	34	21
Paraconexibacter	Thermoleophilia	4	4	7	0
Parahaliea	Gammaproteobacteria	11	15	19	24
Paraherbaspirillum	Betaproteobacteria	2	0	0	1
Paraperlucidibaca	Gammaproteobacteria	0	1	0	2
Pararhizobium	Alphaproteobacteria	0	1	1	2
Pararhodobacter	Alphaproteobacteria	4	0	2	0
Parasediminibacterium	Chitinophagia	4	4	7	3
Parasegetibacter	Chitinophagia	23	2	6	3
Parcubacteriageneraincertaesedis	Parcubacteria	6	0	1	1
Parviterribacter	Thermoleophilia	4	33	22	12
Paucibacter	Betaproteobacteria	19	38	17	14
Paucimonas	Betaproteobacteria	0	1	0	0
Pedococcus	Actinobacteria	0	2	0	0
Pedomicrobium	Alphaproteobacteria	15	17	24	22
Pelagibius	Alphaproteobacteria	0	1	0	0
Pelobacter	Deltaproteobacteria	2	14	5	6
Pelolinea	Anaerolineae	1	7	9	13
Pelomicrobium	Hydrogenophilalia	5	6	8	7
Pelomonas	Betaproteobacteria	3	3	4	3
Peredibacter	Oligoflexia	5	10	12	12
Peribacillus	Bacilli	0	0	1	1

Genus abundance counts	Corresponding class	Site S1	Site S2	Site S3	Site S4
Permianibacter	Gammaproteobacteria	9	15	26	28
Persicirhabdus	Verrucomicrobiae	3	1	4	3
Phaeochromatium	Gammaproteobacteria	0	2	1	0
Phaeodactylibacter	Saprospiria	217	83	119	100
Phaselicystis	Deltaproteobacteria	10	46	40	40
Phenylobacterium	Alphaproteobacteria	1	3	4	2
Phragmitibacter	Verrucomicrobiae	19	11	14	22
Phreatobacter	Alphaproteobacteria	4	10	10	5
Phycicoccus	Actinobacteria	9	5	7	8
Phycisphaera	Phycisphaerae	2	6	6	9
Phyllonema	Cyanobacteria	11	0	1	0
Pirellula	Planctomycetacia	121	102	95	143
Piscinibacter	Betaproteobacteria	31	52	33	23
Piscinibacterium	Betaproteobacteria	16	36	31	26
Piscirickettsia	Gammaproteobacteria	0	0	0	1
Planctomicrobium	Planctomycetacia	42	27	21	37
Planctopirus	Planctomycetacia	0	1	1	1
Planifilum	Bacilli	0	1	0	0
Planktothricoides	Cyanobacteria	3	0	0	1
Pleomorphochaeta	Spirochaetia	0	0	2	0
Plesiocystis	Deltaproteobacteria	2	4	7	10
Plesiomonas	Gammaproteobacteria	0	1	0	0
Polaromonas	Betaproteobacteria	46	34	20	5
Polyangium	Deltaproteobacteria	3	8	7	11
Polymorphobacter	Alphaproteobacteria	6	1	6	1
Polynucleobacter	Betaproteobacteria	1	9	2	4
Porphyrobacter	Alphaproteobacteria	11	7	6	4
Portibacter	Saprospiria	134	57	77	74
Porticoccus	Gammaproteobacteria	0	4	8	9
Poseidonibacter	Campylobacteria	0	1	1	0
Povalibacter	Gammaproteobacteria	237	288	356	308
Prolixibacter	Bacteroidia	0	1	3	1
Propionivibrio	Betaproteobacteria	6	18	12	5
Prosthecobacter	Verrucomicrobiae	18	13	10	10
Prosthecomicrobium	Alphaproteobacteria	1	2	0	2
Pseudacidovorax	Betaproteobacteria	0	3	1	0
Pseudaeromonas	Gammaproteobacteria	0	1	3	1
Pseudarcobacter	Campylobacteria	0	14	1	0
Pseudarthrobacter	Actinobacteria	1	2	5	1
Pseudenhygromyxa	Deltaproteobacteria	5	8	11	9
Pseudobacter	Chitinophagia	1	1	1	0
Pseudobacteriovorax	Oligoflexia	0	0	1	1
Pseudoflavitalea	Chitinophagia	4	0	0	0
Pseudofulvimonas	Gammaproteobacteria	0	0	1	1
Pseudohaliea	Gammaproteobacteria	0	5	4	4
Pseudohalioglobus	Gammaproteobacteria	0	1	0	2
Pseudohalocynthiibacter	Alphaproteobacteria	0	1	0	0
Pseudohongiella	Gammaproteobacteria	2	3	4	11
Pseudolabrys	Alphaproteobacteria	4	47	39	28

Genus abundance counts	Corresponding class	Site S1	Site S2	Site S3	Site S4
Pseudomaricurvus	Gammaproteobacteria	0	1	1	1
Pseudomonas	Gammaproteobacteria	2	15	12	13
Pseudonocardia	Actinobacteria	1	0	2	1
Pseudorhodobacter	Alphaproteobacteria	1	0	0	1
Pseudorhodoferax	Betaproteobacteria	5	7	0	3
Pseudorhodoplanes	Alphaproteobacteria	13	50	50	36
Pseudoruegeria	Alphaproteobacteria	0	0	1	0
Pseudoxanthobacter	Alphaproteobacteria	0	1	0	0
Pseudoxanthomonas	Gammaproteobacteria	0	2	0	0
Puia	Chitinophagia	0	0	0	2
Puzihella	Actinobacteria	0	1	0	2
Pyruvatibacter	Alphaproteobacteria	1	3	0	1
Pyxidicoccus	Deltaproteobacteria	7	8	3	8
Qipengyuania	Alphaproteobacteria	1	2	2	0
Quadrisphaera	Actinobacteria	1	0	1	1
Quisquiliibacterium	Betaproteobacteria	4	5	3	2
Racemicystis	Deltaproteobacteria	4	10	11	4
Ramlibacter	Betaproteobacteria	26	59	41	24
Raoultibacter	Coriobacteriia	1	1	0	1
Rathayibacter	Actinobacteria	1	0	1	0
Rectinema	Spirochaetia	11	21	8	8
Reichenbachiella	Cytophagia	0	0	1	1
Reyranella	Alphaproteobacteria	6	20	9	7
Rheinheimera	Gammaproteobacteria	2	4	7	3
Rhizobacter	Betaproteobacteria	120	182	125	93
Rhizobium	Alphaproteobacteria	1	0	1	0
Rhizomicrobium	Alphaproteobacteria	13	8	10	14
Rhizorhabdus	Alphaproteobacteria	0	6	4	0
Rhizorhapis	Alphaproteobacteria	0	4	0	0
Rhodobacter	Alphaproteobacteria	100	54	54	37
Rhodobium	Alphaproteobacteria	3	0	4	19
Rhodocyclus	Betaproteobacteria	1	2	2	1
Rhodoferax	Betaproteobacteria	58	155	130	37
Rhodoligotrophos	Alphaproteobacteria	8	3	9	5
Rhodoluna	Actinobacteria	1	52	51	10
Rhodopila	Alphaproteobacteria	1	1	2	0
Rhodopirellula	Planctomycetacia	32	11	15	16
Rhodoplanes	Alphaproteobacteria	5	17	19	4
Rhodovastum	Alphaproteobacteria	3	1	2	3
Rhodovulum	Alphaproteobacteria	0	0	2	2
Rickettsia	Alphaproteobacteria	0	3	8	17
Rivicola	Betaproteobacteria	0	1	1	0
Robbsia	Betaproteobacteria	8	6	5	4
Romboutsia	Clostridia	0	1	1	1
Roseateles	Betaproteobacteria	115	204	131	101
Roseibacillus	Verrucomicrobiae	4	1	4	1
Roseiflexus	Chloroflexia	3	4	5	3
Roseimarinus	Bacteroidia	4	3	2	4
Roseimaritima	Planctomycetacia	3	0	0	0

Genus abundance counts	Corresponding class	Site S1	Site S2	Site S3	Site S4
Roseimicrobium	Verrucomicrobiae	6	5	8	8
Roseisolibacter	Gemmatimonadetes	140	182	174	165
Roseithermus	Rhodothermia	5	3	6	7
Roseivivax	Alphaproteobacteria	0	0	1	0
Roseomonas	Alphaproteobacteria	13	17	20	17
Roseovarius	Alphaproteobacteria	4	1	5	5
Rouxiella	Gammaproteobacteria	1	0	0	1
Rubidimonas	Saprospiria	0	1	1	1
Rubinisphaera	Planctomycetacia	35	14	12	18
Rubripirellula	Planctomycetacia	9	0	6	7
Rubritalea	Verrucomicrobiae	0	0	1	0
Rubrivivax	Betaproteobacteria	2	2	3	2
Ruficoccus	Opitutae	0	1	1	3
Runella	Cytophagia	1	0	2	1
Rurimicrobium	Chitinophagia	3	2	3	2
Saccharibacteriageneraincertaesedis	Saccharibacteria	136	64	130	120
Saccharicenans	Saccharicenantaceae	5	18	9	13
Saccharofermentans	Clostridia	0	0	1	0
Saccharophagus	Gammaproteobacteria	0	1	1	1
Salibacter	Flavobacteriia	3	4	16	11
Salinarimonas	Alphaproteobacteria	0	0	2	0
Salinibacterium	Actinobacteria	0	0	4	1
Saliniramus	Alphaproteobacteria	0	0	0	1
Salinirepens	Flavobacteriia	1	3	14	18
Salinispira	Spirochaetia	0	0	1	0
Salipiger	Alphaproteobacteria	0	0	2	0
Sandaracinus	Deltaproteobacteria	18	51	47	65
Sandarakinorhabdus	Alphaproteobacteria	1	0	0	1
Schlegelella	Betaproteobacteria	0	1	1	0
Schlesneria	Planctomycetacia	12	4	8	17
Sediminibacterium	Chitinophagia	8	2	2	5
Segetibacter	Chitinophagia	2	0	0	0
Seohaeicola	Alphaproteobacteria	2	1	1	2
Seongchinamella	Gammaproteobacteria	3	3	5	5
Shewanella	Gammaproteobacteria	0	0	1	1
Shinella	Alphaproteobacteria	1	0	0	0
Sideroxydans	Betaproteobacteria	23	180	51	60
Silanimonas	Gammaproteobacteria	3	0	0	1
Silvanigrella	Oligoflexia	2	3	4	2
Silvibacterium	Acidobacteria_Gp1	1	2	1	0
Simplicispira	Betaproteobacteria	3	7	1	1
Singulisphaera	Planctomycetacia	2	3	0	1
Sinimarinibacterium	Gammaproteobacteria	0	0	0	3
Skermanella	Alphaproteobacteria	0	3	1	2
Smithella	Deltaproteobacteria	11	28	9	11
Solirubrobacter	Thermoleophilia	1	3	2	1
Solitalea	Sphingobacteriia	1	3	5	10
Soortia	Balneolia	1	1	2	1
Sorangium	Deltaproteobacteria	7	2	4	4

Genus abundance counts	Corresponding class	Site S1	Site S2	Site S3	Site S4
Spartobacteriageneraincertaesedis	Spartobacteria	212	132	179	132
Sphaerobacter	Thermomicrobia	0	1	2	0
Sphaerotilus	Betaproteobacteria	2	10	5	2
Sphingoaurantiacus	Alphaproteobacteria	0	0	0	1
Sphingobium	Alphaproteobacteria	0	3	2	2
Sphingomonas	Alphaproteobacteria	45	29	29	19
Sphingopyxis	Alphaproteobacteria	2	1	0	1
Sphingorhabdus	Alphaproteobacteria	277	85	137	30
Spirochaeta	Spirochaetia	1	0	1	1
Sporichthya	Actinobacteria	8	7	6	11
Sporocytophaga	Cytophagia	0	0	0	1
SR1generaincertaesedis	SR1	2	2	3	1
Starkeya	Alphaproteobacteria	2	1	1	0
Stella	Alphaproteobacteria	0	0	2	2
Stenotrophobacter	Blastocatellia	52	40	36	46
Stenotrophobium	Gammaproteobacteria	8	10	4	9
Stenotrophomonas	Gammaproteobacteria	0	1	0	0
Steroidobacter	Gammaproteobacteria	32	76	50	38
Sterolibacterium	Betaproteobacteria	5	11	9	12
Stigmatella	Deltaproteobacteria	0	1	0	2
Streptomyces	Actinobacteria	0	1	1	0
Streptophyta	Chloroplast	0	0	2	0
Subdivision3generaincertaesedis	Verrucomicrobia SD3	342	429	434	500
Subdivision5generaincertaesedis	Verrucomicrobia SD5	11	7	21	14
Sulfuricaulis	Gammaproteobacteria	71	126	55	48
Sulfuricella	Betaproteobacteria	3	21	6	4
Sulfuricurvum	Campylobacteria	1	4	0	1
Sulfuriferula	Betaproteobacteria	0	2	1	0
Sulfuriflexus	Gammaproteobacteria	2	2	1	1
Sulfurirhabdus	Betaproteobacteria	4	20	9	6
Sulfurisoma	Betaproteobacteria	64	107	46	63
Sulfuritalea	Betaproteobacteria	49	157	105	100
Sulfuritortus	Betaproteobacteria	67	62	63	57
Sulfurivermis	Gammaproteobacteria	3	3	2	0
Sunxiuqinia	Bacteroidia	3	7	1	2
Syntrophobacter	Deltaproteobacteria	8	6	5	12
Syntrophorhabdus	Deltaproteobacteria	27	14	17	17
Syntrophus	Deltaproteobacteria	6	22	12	12
Tabrizicola	Alphaproteobacteria	36	18	9	10
Tagaea	Alphaproteobacteria	1	1	0	1
Tahibacter	Gammaproteobacteria	1	0	1	1
Tangfeifania	Bacteroidia	2	3	3	3
Taonella	Alphaproteobacteria	0	2	5	12
Tardiphaga	Alphaproteobacteria	2	4	2	4
Telluribacter	Cytophagia	0	0	0	1
Telmatocola	Planctomycetacia	9	12	8	15
Telmatospirillum	Alphaproteobacteria	1	1	0	3
Temperatibacter	Alphaproteobacteria	0	0	1	0
Tepidicella	Betaproteobacteria	0	2	0	0

Genus abundance counts	Corresponding class	Site S1	Site S2	Site S3	Site S4
Tepidimonas	Betaproteobacteria	1	5	2	1
Tepidisphaera	Phycisphaerae	4	5	6	8
Terracidiphilus	Acidobacteria_Gp1	1	2	2	0
Terrimicrobium	Terrimicrobia	13	26	22	12
Terrimonas	Chitinophagia	326	121	156	168
Tetrasphaera	Actinobacteria	0	1	2	3
Thalassiella	Actinobacteria	1	0	2	0
Thalassocella	Gammaproteobacteria	0	0	1	1
Thalassocola	Alphaproteobacteria	0	0	1	1
Thauera	Betaproteobacteria	20	18	12	6
Thermacetogenium	Clostridia	0	0	1	1
Thermanaerothrix	Anaerolineae	23	34	37	51
Thermoanaerobaculum	Thermoanaerobaculia	1	3	2	1
Thermodesulfitimonas	Clostridia	0	29	1	1
Thermodesulforhabdus	Deltaproteobacteria	0	1	0	0
Thermodesulfovibrio	Nitrospira	6	4	3	9
Thermoflexibacter	Cytophagia	1	0	1	1
Thermoflexus	Thermoflexia	3	51	30	17
Thermogladius	Thermoprotei	4	1	3	3
Thermogutta	Planctomycetacia	14	20	17	18
Thermohalobacter	Clostridia	0	0	1	0
Thermoleophilum	Thermoleophilia	0	1	1	1
Thermomarinilinea	Anaerolineae	21	34	46	44
Thermomonas	Gammaproteobacteria	14	20	11	6
Thermosinus	Negativicutes	1	1	1	1
Thermostilla	Planctomycetacia	57	55	67	150
Thermosulfidibacter	Aquificae	0	1	0	0
Thioalbus	Gammaproteobacteria	36	9	11	20
Thiobacillus	Betaproteobacteria	77	64	22	26
Thiobacter	Betaproteobacteria	28	188	121	117
Thioclava	Alphaproteobacteria	0	0	0	1
Thiococcus	Gammaproteobacteria	1	2	2	2
Thiofaba	Gammaproteobacteria	0	1	0	3
Thioflavicoccus	Gammaproteobacteria	0	0	0	2
Thiogranum	Gammaproteobacteria	6	8	8	7
Thiohalobacter	Gammaproteobacteria	19	18	30	22
Thiohalocapsa	Gammaproteobacteria	1	3	1	2
Thiohalomonas	Gammaproteobacteria	4	6	18	9
Thiohalophilus	Gammaproteobacteria	1	0	0	0
Thiolapillus	Gammaproteobacteria	2	0	0	0
Thioprofundum	Gammaproteobacteria	7	19	21	16
Thiorhodospira	Gammaproteobacteria	0	2	3	0
Tianweitania	Alphaproteobacteria	2	0	2	3
Tibeticola	Betaproteobacteria	1	2	1	0
Tistlia	Alphaproteobacteria	0	2	2	1
Tolumonas	Gammaproteobacteria	0	4	3	0
Treponema	Spirochaetia	3	7	6	3
Truepera	Deinococci	10	3	8	9
Turneriella	Spirochaetia	8	6	7	9

Genus abundance counts	Corresponding class	Site S1	Site S2	Site S3	Site S4
Tuwongella	Planctomycetacia	2	1	2	4
Uliginosibacterium	Betaproteobacteria	0	6	4	4
Unassigned		3623	4168	4117	4537
Undibacterium	Betaproteobacteria	2	2	1	2
Vampirovibrio	Deltaproteobacteria	11	6	4	5
Variibacter	Alphaproteobacteria	4	9	11	3
Variovorax	Betaproteobacteria	41	26	10	8
Verrucomicrobium	Verrucomicrobiae	2	5	3	7
Vicinamibacter	Vicinamibacteria	8	12	13	9
Vicingus	Flavobacteriia	3	5	3	7
Vitiosangium	Deltaproteobacteria	1	10	10	14
Vitreoscilla	Betaproteobacteria	0	1	0	0
Vogesella	Betaproteobacteria	1	5	2	3
Vulcaniibacterium	Gammaproteobacteria	6	0	1	0
Vulgatibacter	Deltaproteobacteria	1	4	6	4
Wagnerdoeblera	Alphaproteobacteria	1	5	1	1
Wandonia	Flavobacteriia	1	2	2	1
Wenyingzhuangia	Flavobacteriia	3	0	0	1
Wenzhouxiangella	Gammaproteobacteria	18	36	55	48
Williamwhitmania	Bacteroidia	0	2	0	1
WoesearchaeotaIncertaeSedisAR15	Woesearchaeota	6	5	5	2
WoesearchaeotaIncertaeSedisAR16	Woesearchaeota	17	28	38	21
WoesearchaeotaIncertaeSedisAR18	Woesearchaeota	0	0	1	1
WoesearchaeotaIncertaeSedisAR20	Woesearchaeota	0	0	2	2
Woeseia	Gammaproteobacteria	118	208	189	284
Wolinella	Campylobacteria	1	0	1	0
Woodsholea	Alphaproteobacteria	2	0	0	0
WPS1generaincertaesedis	WPS-1	13	28	19	23
WPS2generaincertaesedis	WPS-2	0	0	0	1
Xanthobacter	Alphaproteobacteria	1	1	1	0
Xanthomonas	Gammaproteobacteria	2	1	2	1
Xenophilus	Betaproteobacteria	1	5	2	0
Yoonia	Alphaproteobacteria	0	2	0	0
Zavarzinella	Planctomycetacia	25	12	16	17
Zavarzinia	Alphaproteobacteria	1	0	0	0
Zhizhongheella	Betaproteobacteria	19	30	26	31
Zoogloea	Betaproteobacteria	0	3	3	0
