# Supplementary material for: Light Penetration, Dissolved Oxygen, and Riparian Zone Extent Are Associated with the Structure, Gene Expression, and Lipid Production of Riverbed Microbial Communities
Source: Microorganisms. 2026 Oct 21;14(10):2406. doi: 10.3390/microorganisms14102406 (PMC13707542; doi:10.3390/microorganisms14102406)
Supplement: Supplementary file 1 [file microorganisms-14-02406-s001.zip › Supplementary Table S2. Cumulative table of L3-level expressed functions.pdf]

SEED L3	SEED L2	SEED L1	Site S1	Site S2	Site S3	Site S4
(GlcNAc)2_Catabolic_Operon	Aminosugars	Carbohydrates	137	61	82	76
2-Ketogluconate_Utilization	Monosaccharides	Carbohydrates	44	12	10	10
2-phosphoglycolate_salvage	DNA repair	DNA Metabolism	82	98	39	83
4-Hydroxyphenylacetic_acid_catabolic_pathway	Metabolism of central aromatic intermediates	Metabolism of Aromatic Compounds	256	147	203	340
5-FCL-like_protein	Folate and pterines	Cofactors, Vitamins, Prosthetic Groups, Pigments	3799	4559	2945	3242
A_Gammaproteobacteria_Cluster_Relating_to_Translation	Ribosome-related cluster	Clustering-based subsystems	131	169	120	150
A_Glutathione-dependent_Thiol_Reductase_Associated_with_a_Step_in_Lysine_Biosynthesis	Lysine Biosynthesis	Clustering-based subsystems	31	74	20	20
A_Gram-positive_cluster_that_relates_ribosomal_protein_L28P_to_a_set_of_uncharacterized_proteins	Ribosomal Protein L28P relates to a set of uncharac	Clustering-based subsystems	1041	784	666	1054
A_Hypothetical_Protein_Related_to_Proline_Metabolism	Proline and 4-hydroxyproline	Amino Acids and Derivatives	178	149	247	178
ABC_transporter_alkylphosphonate_(TC_3.A.1.9.1)	ABC transporters	Membrane Transport	592	243	248	398
ABC_transporter_branched-chain_amino_acid_(TC_3.A.1.4.1)	ABC transporters	Membrane Transport	7115	5404	4134	4440
ABC_transporter_dipeptide_(TC_3.A.1.5.2)	ABC transporters	Membrane Transport	3507	2946	2506	2607
ABC_transporter_of_unknown_substrate_X	ABC transporters	Membrane Transport	7	6	10	10
ABC_transporter_oligopeptide_(TC_3.A.1.5.1)	ABC transporters	Membrane Transport	4348	3415	25412	3556
ABC_transporter_peptide_(TC_3.A.1.5.5)	ABC transporters	Membrane Transport	63	60	80	70
ABC_transporter_tungstate_(TC_3.A.1.6.2)	ABC transporters	Membrane Transport	61	152	40	79
Accessory_colonization_factor	Adhesion	Virulence, Disease and Defense	26	7	6	6
Acetoin,_butanediol_metabolism	Fermentation	Carbohydrates	358	207	148	261
Acetone_Butanol_Ethanol_Synthesis	Fermentation	Carbohydrates	2	4	3	2
Acetyl-CoA_fermentation_to_Butyrate	Fermentation	Carbohydrates	16268	12942	9392	11803
Acid_resistance_mechanisms	Acid stress	Stress Response	416	263	306	245
Acyclic_terpenes_utilization	Isoprenoids	Fatty Acids, Lipids, and Isoprenoids	240	170	220	160
Acyl-CoA_thioesterase_II	Fatty acids	Fatty Acids, Lipids, and Isoprenoids	92	25	20	20
Adaptation_to_d-cysteine	Resistance to antibiotics and toxic compounds	Virulence, Disease and Defense	43	36	75	25
Additional_flagellar_genes_in_Vibrionales	Flagellar motility in Prokaryota	Motility and Chemotaxis	15	12	10	29
Adenosyl_nucleosidases	Unknown	Nucleosides and Nucleotides	1	1	1	4
Adhesins_in_Staphylococcus	Adhesion	Virulence, Disease and Defense	519	541	387	718
Agrobacterium_opine_transport	Unknown	Membrane Transport	11	3	2	2
Alanine_biosynthesis	Alanine, serine, and glycine	Amino Acids and Derivatives	2224	1924	1169	1889
Alcaligin_Siderophore	Siderophores	Iron acquisition and metabolism	7	18	5	5
Alginate_metabolism	Capsular and extracellular polysacchrides	Cell Wall and Capsule	264	291	274	230
Alkaloid_biosynthesis_from_L-lysine	Plant Alkaloids	Secondary Metabolism	488	187	286	255
Alkanesulfonate_assimilation	Organic sulfur assimilation	Sulfur Metabolism	2040	1885	1399	1458
Alkanesulfonates_Utilization	Organic sulfur assimilation	Sulfur Metabolism	10	8	8	17
Alkylphosphonate_utilization	Unknown	Phosphorus Metabolism	216	140	143	135
Allantoin_Utilization	Unknown	Nitrogen Metabolism	353	457	434	206
Alpha-acetolactate_operon	Organic acids	Carbohydrates	15	12	10	49
Alpha-Amylase_locus_in_Streptocococcus	Polysaccharides	Carbohydrates	755	483	746	546
Amidase_clustered_with_urea_and_nitrile_hydratase_functions	Unknown	Nitrogen Metabolism	21	13	10	31
Aminopeptidases_(EC_3.4.11.-)	Protein degradation	Protein Metabolism	1238	1092	862	850
Ammonia_assimilation	Unknown	Nitrogen Metabolism	3988	3987	2610	3369
AMP_to_3-phosphoglycerate	Unknown	Nucleosides and Nucleotides	28	3	2	2
Anaerobic_benzoate_metabolism	Anaerobic degradation of aromatic compounds	Metabolism of Aromatic Compounds	109	125	147	148
Anaerobic_Oxidative_Degradation_of_L-Ornithine	Arginine; urea cycle, polyamines	Amino Acids and Derivatives	9	48	6	6
Anaerobic_respiratory_reductases	Electron accepting reactions	Respiration	8552	12404	6703	7852
Archaeal_Flagellum	Flagellar motility in Prokaryota	Motility and Chemotaxis	38	11	10	10
Archease	Unknown	Miscellaneous	1	1	2	1

SEED L3	SEED L2	SEED L1	Site S1	Site S2	Site S3	Site S4
Archease2	Unknown	Miscellaneous	1	1	1	4
Arginine_and_Ornithine_Degradation	Arginine; urea cycle, polyamines	Amino Acids and Derivatives	1055	866	712	705
Arginine_Biosynthesis_extended	Arginine; urea cycle, polyamines	Amino Acids and Derivatives	5189	4979	3922	5353
Arginine_Deiminase_Pathway	Arginine; urea cycle, polyamines	Amino Acids and Derivatives	278	324	273	245
Aromatic_Amin_Catabolism	Unknown	Metabolism of Aromatic Compounds	188	178	166	232
Aromatic_amino_acid_degradation	Aromatic amino acids and derivatives	Amino Acids and Derivatives	840	843	755	758
Aromatic_amino_acid_interconversions_with_aryl_acids	Aromatic amino acids and derivatives	Amino Acids and Derivatives	1462	1407	948	916
Arsenic_resistance	Resistance to antibiotics and toxic compounds	Virulence, Disease and Defense	181	396	286	158
At2g33980_At1g28960	Plant-Prokaryote DOE project	Miscellaneous	306	264	161	364
At3g21300	Plant-Prokaryote DOE project	Miscellaneous	42	47	58	78
ATP-dependent_efflux_pump_transporter_Ybh	ABC transporters	Membrane Transport	237	159	207	126
ATP-dependent_RNA_helicases,_bacterial	RNA processing and modification	RNA Metabolism	7354	4902	5738	5288
Autoinducer_2_(AI-2)_transport_and_processing_(lsrACDBFGE_operon)	Quorum sensing and biofilm formation	Regulation and Cell signaling	123	197	114	138
Auxin_biosynthesis	Plant Hormones	Secondary Metabolism	45	23	25	28
Bacillibactin_Siderophore	Siderophores	Iron acquisition and metabolism	119	54	27	82
Bacillus_biofilm_matrix_protein_component_TasA_and_homologs	Unknown	Dormancy and Sporulation	34	30	103	22
Bacitracin_Stress_Response	Bacteriocins, ribosomally synthesized antibacterial	Virulence, Disease and Defense	151	167	117	137
Bacterial_Cell_Division	Unknown	Clustering-based subsystems	11650	9842	8151	8407
Bacterial_Chemotaxis	Unknown	Motility and Chemotaxis	3545	3489	2142	2383
Bacterial_cyanide_production_and_tolerance_mechanisms	Unknown	Virulence, Disease and Defense	80	33	64	88
Bacterial_Cytoskeleton	Unknown	Cell Division and Cell Cycle	7634	6414	5775	6692
Bacterial_hemoglobins	Unknown	Stress Response	1105	951	729	1177
Bacterial_light-harvesting_proteins	Light-harvesting complexes	Photosynthesis	113	41	80	40
Bacterial_RNA-metabolizing_Zn-dependent_hydrolases	Unknown	Clustering-based subsystems	6139	3235	3222	3569
Benzoate_catabolism	Peripheral pathways for catabolism of aromatic cor	Metabolism of Aromatic Compounds	66	75	93	98
Benzoate_degradation	Peripheral pathways for catabolism of aromatic cor	Metabolism of Aromatic Compounds	154	89	76	166
Benzoate_transport_and_degradation_cluster	Unknown	Metabolism of Aromatic Compounds	1401	1081	966	1412
Beta-Glucoside_Metabolism	Di- and oligosaccharides	Carbohydrates	537	755	347	776
Beta-lactamase	Resistance to antibiotics and toxic compounds	Virulence, Disease and Defense	488	513	605	548
Biflavanoid_biosynthesis	Biosynthesis of phenylpropanoids	Secondary Metabolism	29	63	47	22
Bilin_Biosynthesis	Tetrapyrroles	Cofactors, Vitamins, Prosthetic Groups, Pigments	302	104	47	46
Biofilm_Adhesin_Biosynthesis	Quorum sensing and biofilm formation	Regulation and Cell signaling	20	20	13	38
Biofilm_formation_in_Staphylococcus	Quorum sensing and biofilm formation	Regulation and Cell signaling	159	95	141	147
Biogenesis_of_cbb3-type_cytochrome_c_oxidases	Unknown	Respiration	33	70	22	29
Biogenesis_of_c-type_cytochromes	Unknown	Respiration	1222	886	578	902
Biogenesis_of_cytochrome_c_oxidases	Unknown	Respiration	923	709	488	634
Biotin_biosynthesis	Biotin	Cofactors, Vitamins, Prosthetic Groups, Pigments	4921	3807	3079	3292
Biphenyl_Degradation	Peripheral pathways for catabolism of aromatic cor	Metabolism of Aromatic Compounds	39	20	31	49
BlaR1_Family_Regulatory_Sensor-transducer_Disambiguation	Resistance to antibiotics and toxic compounds	Virulence	737	687	538	655
Branched_chain_amino_acid_degradation_regulons	Branched-chain amino acids	Amino Acids and Derivatives	12	16	24	14
Branched-Chain_Amino_Acid_Biosynthesis	Branched-chain amino acids	Amino Acids and Derivatives	13306	12416	9024	10305
Broadly_distributed_proteins_not_in_subsystems	Unknown	Miscellaneous	956	863	659	861
Butanol_Biosynthesis	Fermentation	Carbohydrates	2	26	5	2
C_jejuni_colonization_of_chick_caeca	Unknown	Virulence, Disease and Defense	18	5	4	4
Cadmium_resistance	Resistance to antibiotics and toxic compounds	Virulence, Disease and Defense	123	104	80	100
Caffeic_acid_derivatives	Biosynthesis of phenylpropanoids	Secondary Metabolism	1	3	1	1
Calvin-Benson_cycle	CO2 fixation	Carbohydrates	4417	2619	1792	2948

SEED L3	SEED L2	SEED L1	Site S1	Site S2	Site S3	Site S4
cAMP_signaling_in_bacteria	Unknown	Regulation and Cell signaling	4758	3707	3107	3868
Campylobacter_Iron_Metabolism	Unknown	Iron acquisition and metabolism	72	31	41	36
Capsular_heptose_biosynthesis	Capsular and extracellular polysacchrides	Cell Wall and Capsule	1043	1160	769	938
Capsular_Polysaccharide_(CPS)_of_Campylobacter	Capsular and extracellular polysacchrides	Cell Wall and Capsule	1	1	1	2
Capsular_Polysaccharides_Biosynthesis_and_Assembly	Capsular and extracellular polysacchrides	Cell Wall and Capsule	201	114	166	117
carbazol_degradation_cluster	Unknown	Metabolism of Aromatic Compounds	16	13	12	36
Carbon_monoxide_dehydrogenase_maturation_factors	Unknown	Respiration	60	34	57	72
Carbon_monoxide_induced_hydrogenase	Unknown	Respiration	846	1003	433	315
Carbon_Starvation	Unknown	Stress Response	1271	1105	813	978
Carboxysome	CO2 fixation	Carbohydrates	525	32	59	55
Carnitine_Metabolism_in_Microorganisms	Fatty acids	Fatty Acids, Lipids, and Isoprenoids	17	14	31	14
Carotenoids	Isoprenoids	Fatty Acids, Lipids, and Isoprenoids	1272	1244	624	982
Catechol_branch_of_beta-ketoadipate_pathway	Metabolism of central aromatic intermediates	Metabolism of Aromatic Compounds	1046	987	986	656
CBSS-138119.3.peg.2719	Unknown	Clustering-based subsystems	7900	4532	4049	5369
CBSS-160492.1.peg.550	Unknown	Clustering-based subsystems	56	77	151	41
CBSS-176279.3.peg.1262	Unknown	Clustering-based subsystems	16	13	10	50
CBSS-176279.3.peg.868	Unknown	Clustering-based subsystems	3029	1728	2171	2180
CBSS-176280.1.peg.1561	Unknown	Clustering-based subsystems	123	53	80	78
CBSS-176299.4.peg.1292	Probably GTP or GMP signaling related	Clustering-based subsystems	797	733	650	595
CBSS-188.1.peg.6170	Sarcosine oxidase	Clustering-based subsystems	240	323	161	228
CBSS-188.1.peg.9880	Three hypotheticals linked to lipoprotein biosynthe	Clustering-based subsystems	75	25	14	53
CBSS-196164.1.peg.1690	Cytochrome biogenesis	Clustering-based subsystems	972	792	742	626
CBSS-196164.1.peg.461	Cytochrome biogenesis	Clustering-based subsystems	87	33	65	39
CBSS-196620.1.peg.2477	Unknown	Clustering-based subsystems	47	37	68	74
CBSS-198094.1.peg.4426	Recombination related cluster	Clustering-based subsystems	21	11	9	23
CBSS-203122.12.peg.188	Transposable elements	Phages, Prophages, Transposable elements, Plasmids	154	183	132	215
CBSS-205922.3.peg.1809	Urate degradation	Clustering-based subsystems	257	300	124	136
CBSS-214092.1.peg.3450	Unknown	Clustering-based subsystems	87	169	97	56
CBSS-216592.1.peg.3534	CRISPRs and associated hypotheticals	Clustering-based subsystems	17	6	18	5
CBSS-222523.1.peg.1311	Nucleotidyl-phosphate metabolic cluster	Clustering-based subsystems	1	1	4	1
CBSS-224911.1.peg.435	Unknown	Clustering-based subsystems	645	611	698	800
CBSS-235.1.peg.567	Unknown	Clustering-based subsystems	147	37	82	30
CBSS-243265.1.peg.198	Translation	Clustering-based subsystems	148	121	84	180
CBSS-246196.1.peg.364	Fatty acid metabolic cluster	Clustering-based subsystems	116	74	110	91
CBSS-251221.1.peg.1863	Unknown	Clustering-based subsystems	329	164	101	185
CBSS-257314.1.peg.676	Two related proteases	Clustering-based subsystems	495	373	183	391
CBSS-257314.1.peg.752	Unknown	Clustering-based subsystems	49	69	42	135
CBSS-258594.1.peg.3339	Biosynthesis of galactoglycans and related lipopoly	Clustering-based subsystems	1220	1328	814	838
CBSS-261594.1.peg.2640	Unknown	Clustering-based subsystems	25	20	47	19
CBSS-262316.1.peg.2929	Catabolism of an unknown compound	Clustering-based subsystems	80	68	65	102
CBSS-262719.3.peg.410	Clustering-based subsystems	Clustering-based subsystems	918	839	560	775
CBSS-265072.7.peg.546	Methylamine utilization	Clustering-based subsystems	148	121	52	105
CBSS-266117.6.peg.2476	Unknown	Clustering-based subsystems	1	1	1	2
CBSS-269482.1.peg.1294	Probably organic hydroperoxide resistance related	Clustering-based subsystems	17	14	11	33
CBSS-269799.3.peg.2220	Molybdopterin oxidoreductase	Clustering-based subsystems	1760	1009	1333	2098
CBSS-269801.1.peg.1715	Unknown	Clustering-based subsystems	40	84	28	36
CBSS-269801.1.peg.1725	Unknown	Clustering-based subsystems	12	10	50	8

SEED L3	SEED L2	SEED L1	Site S1	Site S2	Site S3	Site S4
CBSS-281090.3.peg.464	Unknown	Clustering-based subsystems	15	12	37	19
CBSS-288681.3.peg.1039	Unknown	Clustering-based subsystems	48	38	31	107
CBSS-290633.1.peg.1906	Unknown	Clustering-based subsystems	289	99	111	120
CBSS-291331.3.peg.3674	Unknown	Clustering-based subsystems	549	390	373	334
CBSS-292415.3.peg.2341	Clustering-based subsystems	Clustering-based subsystems	170	88	83	101
CBSS-296591.1.peg.2330	Unknown	Clustering-based subsystems	572	464	383	550
CBSS-312309.3.peg.1965	Unknown	Clustering-based subsystems	4235	2927	2331	2739
CBSS-314269.3.peg.1840	Unknown	Clustering-based subsystems	49	22	32	33
CBSS-316057.3.peg.1308	Putative asociate of RNA polymerase sigma-54 fact	Clustering-based subsystems	702	470	390	614
CBSS-316057.3.peg.3521	Unknown	Clustering-based subsystems	141	809	628	1130
CBSS-316057.3.peg.659	Probably Ybbk-related hypothetical membrane pro	Clustering-based subsystems	1	1	2	1
CBSS-316273.3.peg.227	Unknown	Clustering-based subsystems	89	82	60	143
CBSS-316273.3.peg.2378	Unknown	Clustering-based subsystems	58	82	27	29
CBSS-316273.3.peg.2709	Unknown	Clustering-based subsystems	92	25	20	20
CBSS-316273.3.peg.448	Unknown	Clustering-based subsystems	158	113	141	159
CBSS-316273.3.peg.922	Unknown	Clustering-based subsystems	54	32	82	60
CBSS-316407.3.peg.1371	Hypothetical lipase related to Phosphatidate metab	Clustering-based subsystems	83	154	93	348
CBSS-316407.3.peg.2816	Unknown	Clustering-based subsystems	125	81	39	46
CBSS-320372.3.peg.6046	Unknown	Clustering-based subsystems	478	355	338	434
CBSS-320388.3.peg.3759	Carotenoid biosynthesis	Clustering-based subsystems	66	55	47	130
CBSS-323097.3.peg.2594	Unknown	Clustering-based subsystems	365	250	333	361
CBSS-323098.3.peg.2823	Flagella protein?	Clustering-based subsystems	18	9	2	2
CBSS-323850.3.peg.3142	Chemotaxis, response regulators	Clustering-based subsystems	125	177	108	157
CBSS-323850.3.peg.3269	Hypothetical in Lysine biosynthetic cluster	Clustering-based subsystems	60	46	36	102
CBSS-323850.3.peg.3284	Putative GGDEF domain protein related to agglutin	Clustering-based subsystems	101	150	74	116
CBSS-326442.4.peg.1852	Translation	Clustering-based subsystems	739	864	605	690
CBSS-342610.3.peg.1536	DNA polymerase III epsilon cluster	Clustering-based subsystems	439	329	248	858
CBSS-342610.3.peg.1794	Unknown	Clustering-based subsystems	146	111	88	211
CBSS-342610.3.peg.283	D-tyrosyl-tRNA(Tyr) deacylase (EC 3.1.-.-) cluster	Clustering-based subsystems	93	77	47	30
CBSS-344610.3.peg.2335	Choline bitartrate degradation, putative	Clustering-based subsystems	42	14	11	14
CBSS-345072.3.peg.1318	Unknown	Clustering-based subsystems	347	299	179	279
CBSS-350688.3.peg.1509	Unknown	Clustering-based subsystems	1671	861	1133	1646
CBSS-354.1.peg.2917	TldD cluster	Clustering-based subsystems	460	817	3141	381
CBSS-376686.6.peg.291	Biosynthesis of galactoglycans and related lipopoly	Clustering-based subsystems	2	2	3	6
CBSS-393121.3.peg.1913	Unknown	Clustering-based subsystems	66	63	42	86
CBSS-393121.3.peg.2760	Protein export?	Clustering-based subsystems	3873	1961	1922	2930
CBSS-393124.3.peg.2657	Unknown	Clustering-based subsystems	7	6	13	6
CBSS-393130.3.peg.129	Related to Menaquinone-cytochrome C reductase	Clustering-based subsystems	21	58	14	13
CBSS-393130.3.peg.794	Cell Division	Clustering-based subsystems	7	2	2	2
CBSS-393133.3.peg.2787	Unknown	Clustering-based subsystems	6	1	1	1
CBSS-49338.1.peg.459	Tricarboxylate transporter	Clustering-based subsystems	2056	1574	1123	1173
CBSS-52598.3.peg.2843	alpha-proteobacterial cluster of hypotheticals	Clustering-based subsystems	27	35	18	62
CBSS-562.2.peg.5158_SK3_including	Unknown	Clustering-based subsystems	4	3	2	12
CBSS-56780.10.peg.1536	Unknown	Clustering-based subsystems	64	99	42	35
CBSS-69014.3.peg.2094	Membrane-bound hydrogenase	Clustering-based subsystems	9	3	2	12
CBSS-83331.1.peg.3039	Isoprenoid/cell wall biosynthesis: PREDICTED UNDE	Clustering-based subsystems	2506	1924	1372	2237
CBSS-83332.1.peg.3803	Unknown	Clustering-based subsystems	36	29	23	69

SEED L3	SEED L2	SEED L1	Site S1	Site S2	Site S3	Site S4
CBSS-83333.1.peg.946	Unknown	Clustering-based subsystems	9	7	17	6
CBSS-84588.1.peg.1247	Lysine, threonine, methionine, and cysteine	Clustering-based subsystems	739	987	564	1165
CBSS-87626.3.peg.3639	Unknown	Clustering-based subsystems	823	638	464	496
CBSS-89187.3.peg.2957	tRNA sulfuration	Clustering-based subsystems	27	6	5	10
Cell_Division_Cluster	Cell Division	Clustering-based subsystems	147	205	113	92
Cell_division-ribosomal_stress_proteins_cluster	Unknown	Clustering-based subsystems	1449	988	1052	1471
Cell_envelope-associated_LytR-CpsA-Psr_transcriptional_attenuators	Unknown	Regulation and Cell signaling	33	27	102	25
Cellulosome	Polysaccharides	Carbohydrates	883	761	531	800
Central_meta-cleavage_pathway_of_aromatic_compound_degradation	Metabolism of central aromatic intermediates	Metabolism of Aromatic Compounds	380	390	207	285
Chitin_and_N-acetylglucosamine_utilization	Aminosugars	Carbohydrates	689	651	732	530
Chloroaromatic_degradation_pathway	Peripheral pathways for catabolism of aromatic cor	Metabolism of Aromatic Compounds	7	10	14	6
Chlorophyll_Biosynthesis	Tetrapyrroles	Cofactors, Vitamins, Prosthetic Groups, Pigments	2736	791	2611	1761
Chlorophyll_Degradation	Tetrapyrroles	Cofactors, Vitamins, Prosthetic Groups, Pigments	9	3	4	2
Chlorosome	Light-harvesting complexes	Photosynthesis	5	4	3	10
Cholesterol_catabolic_operon_in_Mycobacteria	Unknown	Fatty Acids, Lipids, and Isoprenoids	6	5	4	20
Choline_and_Betaine_Uptake_and_Betaine_Biosynthesis	Osmotic stress	Stress Response	1377	1284	1049	1138
Choline_Transport	Unknown	Membrane Transport	31	25	20	59
Chorismate:_Intermediate_for_synthesis_of_PAPA_antibiotics,_PABA,_anthranilate,_3-hydroxyanthranilate_	Aromatic amino acids and derivatives	Amino Acids and Derivatives	769	705	367	628
Chorismate_Synthesis	Aromatic amino acids and derivatives	Amino Acids and Derivatives	547	270	204	473
Citrate_Utilization_System_(CitAB,_CitH,_and_tctABC)	Unknown	Membrane Transport	15	24	8	14
Clavulanic_acid_biosynthesis	Bacterial cytostatics, differentiation factors and ant	Secondary Metabolism	7	2	2	2
Cluster_Ytf_and_putative_sugar_transporter	Carbohydrates	Clustering-based subsystems	158	147	126	96
Cluster-based_Subsystem_Grouping_Hypotheticals_-_perhaps_Proteosome_Related	proteosome related	Clustering-based subsystems	223	75	64	111
CMP-N-acetylneuraminate_Biosynthesis	Capsular and extracellular polysacchrides	Cell Wall and Capsule	286	243	203	308
CO_Dehydrogenase	Electron donating reactions	Respiration	1865	1099	1379	1712
CO2_uptake,_carboxysome	CO2 fixation	Carbohydrates	11194	7382	37206	38833
Coagulation_cascade	Proteolytic pathway	Regulation and Cell signaling	91	37	40	70
Cobalamin_synthesis	Tetrapyrroles	Cofactors, Vitamins, Prosthetic Groups, Pigments	47	53	31	30
Cobalt-zinc-cadmium_resistance	Resistance to antibiotics and toxic compounds	Virulence, Disease and Defense	3654	3274	2342	2764
Coenzyme_A_Biosynthesis	Coenzyme A	Cofactors, Vitamins, Prosthetic Groups, Pigments	2595	2177	1741	2150
Coenzyme_B12_biosynthesis	Tetrapyrroles	Cofactors, Vitamins, Prosthetic Groups, Pigments	1791	1359	876	878
Coenzyme_F420_synthesis	Coenzyme F420	Cofactors, Vitamins, Prosthetic Groups, Pigments	87	145	96	177
Coenzyme_F420-H2_dehydrogenase_(methanophenazine)	Electron donating reactions	Respiration	9	9	9	12
coenzyme_M_biosynthesis	Coenzyme M	Cofactors, Vitamins, Prosthetic Groups, Pigments	12	10	10	16
COG2363	Plant-Prokaryote DOE project	Miscellaneous	1682	834	815	744
Colanic_acid_biosynthesis	Capsular and extracellular polysacchrides	Cell Wall and Capsule	67	52	43	127
Cold_shock,_CspA_family_of_proteins	Cold shock	Stress Response	4117	2782	2939	3758
Common_Pathway_For_Synthesis_of_Aromatic_Compounds_(DAHP_synthase_to_chorismate)	Aromatic amino acids and derivatives	Amino Acids and Derivatives	2430	2385	1666	2366
Conserved_cluster_in_Enterobacteriaceae_downstream_from_YqjA,_a_DedA_family_protein	Unknown	Clustering-based subsystems	153	25	20	20
Conserved_gene_cluster_associated_with_Met-tRNA_formyltransferase	Unknown	Clustering-based subsystems	6127	4511	3846	4549
Conserved_gene_cluster_possibly_involved_in_RNA_metabolism	Plant-Prokaryote DOE project	Miscellaneous	74	20	16	16
Control_of_cell_elongation_-_division_cycle_in_Bacilli	Unknown	Cell Division and Cell Cycle	107	64	136	88
Control_of_Swarming_in_Vibrio_and_Shewanella_species	Social motility and nonflagellar swimming in bacter	Motility and Chemotaxis	6	5	4	12
Copper_homeostasis	Resistance to antibiotics and toxic compounds	Virulence, Disease and Defense	3181	2785	2381	2949
Core_Oligosaccharide_Glycosylation_in_Pseudomonas	Gram-Negative cell wall components	Cell Wall and Capsule	40	11	9	9
Creatine_and_Creatinine_Degradation	Unknown	Amino Acids and Derivatives	439	237	206	404
CRISP_Cmr_Cluster	CRISPs	DNA Metabolism	5	4	10	3

SEED L3	SEED L2	SEED L1	Site S1	Site S2	Site S3	Site S4
CRISPRs	CRISPs	DNA Metabolism	161	66	51	113
Curli_production	Protein secretion system, Type VIII (Extracellular nu	Membrane Transport	1	1	1	2
Cyanate_hydrolysis	Unknown	Nitrogen Metabolism	18	42	20	28
Cyanobacterial_Circadian_Clock	Unknown	Cell Division and Cell Cycle	1487	387	483	452
Cyanophycin_Metabolism	Arginine; urea cycle, polyamines	Amino Acids and Derivatives	428	368	197	191
Cysteine_Biosynthesis	Lysine, threonine, methionine, and cysteine	Amino Acids and Derivatives	4141	2868	2286	2993
Cytochrome_B6-F_complex	Unknown	Respiration	1455	620	1763	1803
CytR_regulation	Unknown	Regulation and Cell signaling	12	10	8	24
D-allose_utilization	Monosaccharides	Carbohydrates	54	14	11	14
De_Novo_Purine_Biosynthesis	Purines	Nucleosides and Nucleotides	6346	6189	3866	4786
De_Novo_Pyrimidine_Synthesis	Pyrimidines	Nucleosides and Nucleotides	5910	5154	4384	4653
Dehydrogenase_complexes	Central carbohydrate metabolism	Carbohydrates	14911	14128	9428	9001
Denitrification	Unknown	Nitrogen Metabolism	3779	9612	5051	5913
Deoxyribose_and_Deoxynucleoside_Catabolism	Monosaccharides	Carbohydrates	702	665	504	785
D-galactarate,_D-glucarate_and_D-glycerate_catabolism	Monosaccharides	Carbohydrates	291	514	131	159
D-galactonate_catabolism	Monosaccharides	Carbohydrates	684	197	191	230
D-Galacturonate_and_D-Glucuronate_Utilization	Monosaccharides	Carbohydrates	1547	1023	1209	858
D-gluconate_and_ketogluconates_metabolism	Monosaccharides	Carbohydrates	512	485	272	366
Dihydroxyacetone_kinases	Central carbohydrate metabolism	Carbohydrates	432	491	325	271
Dimethylarginine_metabolism	Unknown	Stress Response	60	200	197	75
Dimethylsulfoniopropionate_(DMSP)_mineralization	Unknown	Sulfur Metabolism	2	14	2	2
Dipeptidases_(EC_3.4.13.-)	Protein degradation	Protein Metabolism	47	91	31	90
Dissimilatory_nitrite_reductase	Unknown	Nitrogen Metabolism	364	492	390	384
DNA_Helicase_of_Unknown_Function	DNA replication	DNA Metabolism	56	16	13	14
DNA_phosphorothioation	Unknown	DNA Metabolism	31	25	20	59
DNA_processing_cluster	DNA uptake, competence	DNA Metabolism	1725	1864	1168	1437
DNA_repair,_bacterial	DNA repair	DNA Metabolism	3998	2988	2741	3077
DNA_repair,_bacterial_DinG_and_relatives	DNA repair	DNA Metabolism	185	143	76	167
DNA_repair,_bacterial_MutL-MutS_system	DNA repair	DNA Metabolism	999	523	314	506
DNA_repair,_bacterial_photolyase	DNA repair	DNA Metabolism	156	109	72	224
DNA_repair,_bacterial_RecBCD_pathway	DNA repair	DNA Metabolism	165	167	166	164
DNA_repair,_bacterial_RecFOR_pathway	DNA repair	DNA Metabolism	841	379	352	656
DNA_repair,_bacterial_UmuCD_system	DNA repair	DNA Metabolism	3826	2215	1604	1980
DNA_repair,_bacterial_UvrD_and_related_helicases	DNA repair	DNA Metabolism	754	890	543	761
DNA_repair,_UvrABC_system	DNA repair	DNA Metabolism	4576	2990	2657	3266
DNA_repair_and_recombination_eukaryotic	DNA repair	DNA Metabolism	31	25	60	20
DNA_Repair_Base_Excision	DNA repair	DNA Metabolism	826	897	656	779
DNA_replication,_archaeal	DNA replication	DNA Metabolism	139	62	100	202
DNA_structural_proteins,_bacterial	Unknown	DNA Metabolism	2907	2175	1749	2256
DNA_topoisomerases,_Type_I,_ATP-independent	DNA replication	DNA Metabolism	455	310	147	492
DNA_topoisomerases,_Type_II,_ATP-dependent	DNA replication	DNA Metabolism	158	84	78	104
DNA-binding_regulatory_proteins,_strays	Unknown	Regulation and Cell signaling	94	249	112	101
DNA-replication	DNA replication	DNA Metabolism	6144	5979	3557	4665
D-ribose_utilization	Monosaccharides	Carbohydrates	1141	35238	17755	6542
D-Sorbitol(D-Glucitol)_and_L-Sorbose_Utilization	Monosaccharides	Carbohydrates	18	15	12	37
D-Tagatose_and_Galactitol_Utilization	Monosaccharides	Carbohydrates	587	427	181	405
dTDP-rhamnose_synthesis	Capsular and extracellular polysacchrides	Cell Wall and Capsule	320	389	301	411

SEED L3	SEED L2	SEED L1	Site S1	Site S2	Site S3	Site S4
EC49-61	Unknown	Clustering-based subsystems	493	213	196	356
EC699-706	Unknown	Clustering-based subsystems	243	351	225	293
ECF_class_transporters	Unknown	Membrane Transport	31	33	38	67
Ectoine_biosynthesis_and_regulation	Osmotic stress	Stress Response	26	23	57	18
Entner-Doudoroff_Pathway	Central carbohydrate metabolism	Carbohydrates	2930	2872	2172	2288
Erythritol_utilization	Sugar alcohols	Carbohydrates	63	46	31	89
ESAT-6_proteins_secretion_system_in_Actinobacteria	Protein translocation across cytoplasmic membran	Membrane Transport	22	6	6	5
ESAT-6_proteins_secretion_system_in_Firmicutes	Protein translocation across cytoplasmic membran	Membrane Transport	2	2	2	5
Ethanolamine_utilization	Sugar alcohols	Carbohydrates	530	361	304	285
Ethylmalonyl-CoA_pathway_of_C2_assimilation	Central carbohydrate metabolism	Carbohydrates	462	441	334	473
eukaryotic_rRNA_modification_and_related_functions	RNA processing and modification	RNA Metabolism	56	22	183	57
Exopolysaccharide_Biosynthesis	Capsular and extracellular polysacchrides	Cell Wall and Capsule	566	475	424	499
Exosporium	Unknown	Dormancy and Sporulation	2	10	2	2
Extracellular_Polysaccharide_Biosynthesis_of_Streptococci	Capsular and extracellular polysacchrides	Cell Wall and Capsule	6	11	4	10
F0F1-type_ATP_synthase	ATP synthases	Respiration	28539	27040	27720	32223
Fatty_Acid_Biosynthesis_FASI	Fatty acids	Fatty Acids, Lipids, and Isoprenoids	163	228	301	221
Fatty_Acid_Biosynthesis_FASII	Fatty acids	Fatty Acids, Lipids, and Isoprenoids	14836	12670	19583	12549
Fatty_acid_degradation_regulons	Fatty acids	Fatty Acids, Lipids, and Isoprenoids	1622	1123	781	845
Fermentations:_Lactate	Fermentation	Carbohydrates	587	521	348	675
Fermentations:_Mixed_acid	Fermentation	Carbohydrates	2506	4225	1521	1221
Ferrous_iron_transporter_EfeUOB,_low-pH-induced	Unknown	Iron acquisition and metabolism	31	25	20	59
Fimbriae	Fimbriae of the Chaperone/Usher Assembly Pathw	Virulence	4	9	2	2
Flagellar_motility	Flagellar motility in Prokaryota	Motility and Chemotaxis	3482	4195	2362	3555
Flagellum	Flagellar motility in Prokaryota	Motility and Chemotaxis	4880	6767	4894	5936
Flagellum_in_Campylobacter	Flagellar motility in Prokaryota	Motility and Chemotaxis	3336	3865	3808	2805
Flavanone_biosynthesis	Biosynthesis of phenylpropanoids	Secondary Metabolism	23	46	18	10
Flavocytochrome_C	Unknown	Respiration	11	9	7	21
Flavodoxin	Riboflavin, FMN, FAD	Cofactors, Vitamins, Prosthetic Groups, Pigments	25	32	42	16
Flavohaemoglobin	Unknown	Stress Response	103	187	58	73
Folate_Biosynthesis	Folate and pterines	Cofactors, Vitamins, Prosthetic Groups, Pigments	2478	1982	1688	2218
Formaldehyde_assimilation:_Ribulose_monophosphate_pathway	One-carbon Metabolism	Carbohydrates	512	357	315	233
Formate_dehydrogenase	Electron donating reactions	Respiration	93	28	31	24
Formate_hydrogenase	Unknown	Respiration	5263	4117	3340	3651
Fructooligosaccharides(FOS)_and_Raffinose_Utilization	Di- and oligosaccharides	Carbohydrates	484	399	284	430
Fructose_and_Mannose_Inducible_PTS	Sugar Phosphotransferase Systems, PTS	Membrane Transport	10	10	6	21
Fructose_utilization	Monosaccharides	Carbohydrates	1345	1219	816	642
GABA_and_putrescine_metabolism_from_cluters	Putrescine/GABA utilization cluster-temporal,to ad	Clustering-based subsystems	262	294	148	146
Galactosylceramide_and_Sulfatide_metabolism	Unknown	Sulfur Metabolism	1751	1090	1092	1170
General_Secretion_Pathway	Protein secretion system, Type II	Membrane Transport	1759	1620	1408	1521
Global_Two-component_Regulator_PrrBA_in_Proteobacteria	Unknown	Regulation and Cell signaling	101	37	26	32
Glutamate_dehydrogenases	Glutamine, glutamate, aspartate, asparagine; amm	Amino Acids and Derivatives	1	3	1	1
Glutamate_transporter_involved_in_acid_tolerance_in_Streptococcus	Acid stress	Stress Response	91	49	40	100
Glutamine,_Glutamate,_Aspartate_and_Asparagine_Biosynthesis	Glutamine, glutamate, aspartate, asparagine; amm	Amino Acids and Derivatives	11923	10531	7858	8370
Glutaredoxins	Oxidative stress	Stress Response	827	403	375	674
Glutathione:_Biosynthesis_and_gamma-glutamyl_cycle	Oxidative stress	Stress Response	699	1100	619	564
Glutathione:_Non-redox_reactions	Oxidative stress	Stress Response	1276	1308	1028	1193
Glutathione:_Redox_cycle	Oxidative stress	Stress Response	584	436	458	432

SEED L3	SEED L2	SEED L1	Site S1	Site S2	Site S3	Site S4
Glutathione_analogs:_mycothiol	Oxidative stress	Stress Response	276	139	113	126
Glutathione-dependent_pathway_of_formaldehyde_detoxification	Detoxification	Stress Response	504	594	330	492
Glutathione-regulated_potassium-efflux_system_and_associated_functions	Unknown	Potassium metabolism	636	847	385	606
Glycerate_metabolism	Organic acids	Carbohydrates	1985	1823	1527	1385
Glycerol_and_Glycerol-3-phosphate_Uptake_and_Utilization	Sugar alcohols	Carbohydrates	3768	2809	19362	2225
Glycerol_fermenation_to_1,3-propanediol	Sugar alcohols	Carbohydrates	12	45	8	8
Glycerolipid_and_Glycerophospholipid_Metabolism_in_Bacteria	Phospholipids	Fatty Acids, Lipids, and Isoprenoids	2487	1901	1401	1980
Glycine_and_Serine_Utilization	Alanine, serine, and glycine	Amino Acids and Derivatives	2571	2422	1509	1771
Glycine_Biosynthesis	Alanine, serine, and glycine	Amino Acids and Derivatives	2375	2181	1501	1747
Glycine_cleavage_system	Alanine, serine, and glycine	Amino Acids and Derivatives	134	76	81	50
Glycine_reductase,_sarcosine_reductase_and_betaine_reductase	Selenoproteins	Protein Metabolism	218	168	211	242
Glycogen_metabolism	Polysaccharides	Carbohydrates	5720	4315	3608	3431
Glycolate,_glyoxylate_interconversions	Central carbohydrate metabolism	Carbohydrates	1361	1080	634	724
Glycolysis_and_Gluconeogenesis	Central carbohydrate metabolism	Carbohydrates	5252	5268	4251	4708
Glycolysis_and_Gluconeogenesis,_including_Archaeal_enzymes	Central carbohydrate metabolism	Carbohydrates	499	517	338	327
Glyoxylate_bypass	Central carbohydrate metabolism	Carbohydrates	1188	1361	1049	919
Gram_Positive_Competence	DNA uptake, competence	DNA Metabolism	4	3	2	17
GroEL_GroES	Protein folding	Protein Metabolism	55819	61819	45719	33879
Group_II_intron-associated_genes	Unknown	RNA Metabolism	7384	79118	36896	27605
H2:CoM-S-S-HTP_oxidoreductase	Electron donating reactions	Respiration	4	9	2	2
Heat_shock_dnaK_gene_cluster_extended	Heat shock	Stress Response	25751	25826	19647	16718
Heme,_hemin_uptake_and_utilization_systems_in_GramPositives	Unknown	Iron acquisition and metabolism	435	307	345	398
Heme_and_Siroheme_Biosynthesis	Tetrapyrroles	Cofactors, Vitamins, Prosthetic Groups, Pigments	2638	2931	1767	2833
Heme_biosynthesis_orphans	Tetrapyrroles	Cofactors, Vitamins, Prosthetic Groups, Pigments	213	69	113	98
Hemin_transport_system	Unknown	Iron acquisition and metabolism	344	151	144	173
Heterocyst_formation_in_cyanobacteria	Unknown	Cell Division and Cell Cycle	20	4	3	3
Hexose_Phosphate_Uptake_System	Monosaccharides	Carbohydrates	64	51	27	52
Hfl_operon	Unknown	Stress Response	1015	848	1153	1037
High_affinity_phosphate_transporter_and_control_of_PHO_regulon	Unknown	Phosphorus Metabolism	25	32	24	29
Histidine_Biosynthesis	Histidine Metabolism	Amino Acids and Derivatives	2840	1968	1538	2119
Histidine_Degradation	Histidine Metabolism	Amino Acids and Derivatives	2030	1774	785	814
HMG_CoA_Synthesis	Branched-chain amino acids	Amino Acids and Derivatives	205	117	191	82
Homogentisate_pathway_of_aromatic_compound_degradation	Metabolism of central aromatic intermediates	Metabolism of Aromatic Compounds	330	178	129	202
Housecleaning_nucleoside_triphosphate_pyrophosphatases	Detoxification	Nucleosides and Nucleotides	428	407	189	148
HPr_catabolite_repression_system	Unknown	Regulation and Cell signaling	510	266	301	306
HtrA_and_Sec_secretion	Protein translocation across cytoplasmic membran	Membrane Transport	121	64	67	113
Hydantoin_metabolism	Unknown	Nucleosides and Nucleotides	1173	967	1015	762
Hydrogenases	Electron donating reactions	Respiration	2120	2895	1520	1744
Hydroxyaromatic_decarboxylase_family	Anaerobic degradation of aromatic compounds	Metabolism of Aromatic Compounds	10	8	6	19
Hyperosmotic_potassium_uptake	Unknown	Potassium metabolism	4	3	22	6
Hypothetical_Related_to_Dihydroorotate_dehydrogenase	Hypothetical Related to Dihydroorate Dehydrogena	Clustering-based subsystems	33	23	7	7
Inner_membrane_protein_YhjD_and_conserved_cluster_involved_in_LPS_biosynthesis	Gram-Negative cell wall components	Cell Wall and Capsule	31	25	20	59
Inorganic_Sulfur_Assimilation	Inorganic sulfur assimilation	Sulfur Metabolism	1576	1207	929	1097
Inositol_catabolism	Sugar alcohols	Carbohydrates	2290	1717	1304	1553
Integrons	Unknown	Phages, Prophages, Transposable elements, Plasmids	32	26	21	62
Inteins	Protein processing and modification	Protein Metabolism	124	46	39	59
Intracellular_septation_in_Enterobacteria	Unknown	Cell Division and Cell Cycle	55	44	67	94

SEED L3	SEED L2	SEED L1	Site S1	Site S2	Site S3	Site S4
Iojap	Plant-Prokaryote DOE project	Miscellaneous	3882	2465	2350	3187
Iron_acquisition_in_Streptococcus	Unknown	Iron acquisition and metabolism	915	1005	693	686
Iron_acquisition_in_Vibrio	Unknown	Iron acquisition and metabolism	26	123	17	110
Isobutyryl-CoA_to_Propionyl-CoA_Module	Organic acids	Carbohydrates	4	11	1	1
Isoleucine_degradation	Branched-chain amino acids	Amino Acids and Derivatives	1254	742	988	1233
Isoprenoid_Biosynthesis	Isoprenoids	Fatty Acids, Lipids, and Isoprenoids	1342	880	739	840
KDO2-Lipid_A_biosynthesis	Gram-Negative cell wall components	Cell Wall and Capsule	4385	3526	2467	3505
Ketoisovalerate_oxidoreductase	Branched-chain amino acids	Amino Acids and Derivatives	33	54	27	55
KH_domain_RNA_binding_protein_YlqC	Unknown	Clustering-based subsystems	781	594	686	797
Lactate_utilization	Organic acids	Carbohydrates	648	1038	478	458
Lacto-N-Biose_I_and_Galacto-N-Biose_Metabolic_Pathway	Unknown	Carbohydrates	7	8	5	16
Lactose_and_Galactose_Uptake_and_Utilization	Di- and oligosaccharides	Carbohydrates	427	418	233	271
Lactose_utilization	Di- and oligosaccharides	Carbohydrates	50	17	14	21
L-Arabinose_utilization	Monosaccharides	Carbohydrates	749	659	560	484
L-ascorbate_utilization_(and_related_gene_clusters)	Monosaccharides	Carbohydrates	7	2	2	2
L-Cystine_Uptake_and_Metabolism	Organic sulfur assimilation	Sulfur Metabolism	2	4	2	3
Legionaminic_Acid_Biosynthesis	Capsular and extracellular polysacchrides	Cell Wall and Capsule	189	140	130	176
Leucine_Biosynthesis	Branched-chain amino acids	Amino Acids and Derivatives	27	22	24	17
Leucine_Degradation_and_HMG-CoA_Metabolism	Branched-chain amino acids	Amino Acids and Derivatives	1960	1801	1615	1629
L-fucose_utilization	Monosaccharides	Carbohydrates	282	162	127	131
L-fucose_utilization_temp	Monosaccharides	Carbohydrates	256	137	110	113
Lipid_A_modifications	Gram-Negative cell wall components	Cell Wall and Capsule	175	180	144	91
Lipid_A-Ara4N_pathway_(_Polymyxin_resistance_)	Gram-Negative cell wall components	Cell Wall and Capsule	547	481	437	602
Lipoic_acid_metabolism	Lipoic acid	Cofactors, Vitamins, Prosthetic Groups, Pigments	1108	442	457	743
Lipopolysaccharide_assembly	Gram-Negative cell wall components	Cell Wall and Capsule	1293	1625	864	938
Lipopolysaccharide-related_cluster_in_Alphaproteobacteria	Gram-Negative cell wall components	Cell Wall and Capsule	74	150	45	78
Lipoprotein_Biosynthesis	Protein processing and modification	Protein Metabolism	38	86	25	42
Lipoprotein_sorting_system	Gram-Negative cell wall components	Cell Wall and Capsule	240	221	251	204
Listeria_Pathogenicity_Island_LIPI-1_extended	Pathogenicity islands	Phages, Prophages, Transposable elements, Plasmids	28	27	55	18
Listeria_surface_proteins:_Internalin-like_proteins	Invasion and intracellular resistance	Virulence, Disease and Defense	885	442	482	863
Listeria_surface_proteins:_LPXTG_motif	Invasion and intracellular resistance	Virulence, Disease and Defense	6	3	2	4
LMPTP_YfkJ_cluster	Unknown	Clustering-based subsystems	109	72	52	131
LMPTP_YwlE_cluster	Unknown	Clustering-based subsystems	512	351	212	280
LOS_core_oligosaccharide_biosynthesis	Gram-Negative cell wall components	Cell Wall and Capsule	592	462	247	386
L-rhamnose_utilization	Monosaccharides	Carbohydrates	542	511	336	377
Luciferases	Unknown	Miscellaneous	7	34	5	5
Lysine_biosynthesis_AAA_pathway_2	Lysine, threonine, methionine, and cysteine	Amino Acids and Derivatives	51	24	19	79
Lysine_Biosynthesis_DAP_Pathway	Lysine, threonine, methionine, and cysteine	Amino Acids and Derivatives	2216	2113	1548	2000
Lysine_degradation	Lysine, threonine, methionine, and cysteine	Amino Acids and Derivatives	1093	1124	748	1042
Lysine_fermentation	Lysine, threonine, methionine, and cysteine	Amino Acids and Derivatives	99	226	123	97
Macromolecular_synthesis_operon	Unknown	Cell Division and Cell Cycle	504	533	723	444
Major_Outer_Membrane_Proteins	Gram-Negative cell wall components	Cell Wall and Capsule	1329	1329	645	781
Malonate_decarboxylase	Organic acids	Carbohydrates	7	6	5	16
Maltose_and_Maltodextrin_Utilization	Di- and oligosaccharides	Carbohydrates	1678	1211	1133	1193
Mannitol_Utilization	Sugar alcohols	Carbohydrates	243	444	114	120
Mannose_Metabolism	Monosaccharides	Carbohydrates	1417	1327	796	1037
Mannose-sensitive_hemagglutinin_type_4_pilus	Protein and nucleoprotein secretion system, Type I	Membrane Transport	315	354	226	441

SEED L3	SEED L2	SEED L1	Site S1	Site S2	Site S3	Site S4
MazEF_toxin-antitoxing_(programmed_cell_death)_system	Programmed Cell Death and Toxin-antitoxin System	Regulation and Cell signaling	113	41	58	48
Mediator_of_hyperadherence_YidE_in_Enterobacteria_and_its_conserved_region	Adhesion	Virulence, Disease and Defense	306	270	334	356
Melibiose_Utilization	Di- and oligosaccharides	Carbohydrates	36	27	76	56
Menaquinone_and_Phylloquinone_Biosynthesis	Quinone cofactors	Cofactors, Vitamins, Prosthetic Groups, Pigments	308	358	178	183
Menaquinone_Biosynthesis_via_Futalosine_--_gjo	Quinone cofactors	Cofactors, Vitamins, Prosthetic Groups, Pigments	425	198	337	454
Mercuric_reductase	Resistance to antibiotics and toxic compounds	Virulence, Disease and Defense	595	285	347	300
Mercury_resistance_operon	Resistance to antibiotics and toxic compounds	Virulence, Disease and Defense	2	2	2	5
Metallocarboxypeptidases_(EC_3.4.17.-)	Protein degradation	Protein Metabolism	316	262	299	173
Metalloendopeptidases_(EC_3.4.24.-)	Protein degradation	Protein Metabolism	130	103	133	188
Methanogenesis	One-carbon Metabolism	Carbohydrates	295	46	38	59
Methanogenesis_from_methylated_compounds	One-carbon Metabolism	Carbohydrates	67	58	30	69
methanopterin_biosynthesis	Folate and pterines	Cofactors, Vitamins, Prosthetic Groups, Pigments	1	1	1	6
Methanopterin_biosynthesis2	Folate and pterines	Cofactors, Vitamins, Prosthetic Groups, Pigments	34	35	80	59
Methionine_Biosynthesis	Lysine, threonine, methionine, and cysteine	Amino Acids and Derivatives	19308	15470	11768	13871
Methionine_Degradation	Lysine, threonine, methionine, and cysteine	Amino Acids and Derivatives	398	456	298	363
Methionine_Salvage	Lysine, threonine, methionine, and cysteine	Amino Acids and Derivatives	177	146	166	276
Methylcitrate_cycle	Organic acids	Carbohydrates	1272	2182	1079	960
Methylglyoxal_Metabolism	Central carbohydrate metabolism	Carbohydrates	2210	1901	1340	1879
Methylthiotransferases	RNA processing and modification	RNA Metabolism	1465	1383	903	1624
MexA-MexB-OprM_Multidrug_Efflux_System	Resistance to antibiotics and toxic compounds	Virulence, Disease and Defense	10	2	5	2
MexE-MexF-OprN_Multidrug_Efflux_System	Resistance to antibiotics and toxic compounds	Virulence, Disease and Defense	134	42	35	80
MLST	Detection	Virulence, Disease and Defense	94	161	78	181
Molybdenum_cofactor_biosynthesis	Folate and pterines	Cofactors, Vitamins, Prosthetic Groups, Pigments	3055	2277	2076	2347
Molybdopterin_cytosine_dinucleotide	Unknown	Cofactors, Vitamins, Prosthetic Groups, Pigments	20	16	35	27
Muconate_lactonizing_enzyme_family	Unknown	Miscellaneous	22	18	21	36
Multidrug_efflux_pump_in_Campylobacter_jejuni_(CmeABC_operon)	Resistance to antibiotics and toxic compounds	Virulence, Disease and Defense	12	14	5	21
Multidrug_Resistance_Efflux_Pumps	Resistance to antibiotics and toxic compounds	Virulence, Disease and Defense	4666	3947	2944	3593
Multidrug_Resistance_Operon_mdtRP_of_Bacillus	Resistance to antibiotics and toxic compounds	Virulence, Disease and Defense	2	2	2	5
Murein_hydrolase_regulation_and_cell_death	Programmed Cell Death and Toxin-antitoxin System	Regulation and Cell signaling	315	186	242	268
Murein_Hydrolases	Unknown	Cell Wall and Capsule	508	865	505	4035
mycolic_acid_synthesis	Cell wall of Mycobacteria	Cell Wall and Capsule	4	11	2	6
Myxoxanthophyll_biosynthesis_in_Cyanobacteria	Isoprenoids	Fatty Acids, Lipids, and Isoprenoids	88	22	43	17
Na(+)_H(+)_antiporter	Uni- Sym- and Antiporters	Membrane Transport	283	200	308	363
Na(+)-translocating_NADH-quinone_oxidoreductase_and_rnf-like_group_of_electron_transport_complexes	Electron donating reactions	Respiration	1138	1045	907	1300
Na+_translocating_decarboxylases_and_related_biotin-dependent_enzymes	Sodium Ion-Coupled Energetics	Respiration	270	298	212	95
N-Acetyl-Galactosamine_and_Galactosamine_Utilization	Aminosugars	Carbohydrates	70	58	55	129
NAD_and_NADP_cofactor_biosynthesis_global	NAD and NADP	Cofactors, Vitamins, Prosthetic Groups, Pigments	1388	1210	989	1295
NAD_regulation	NAD and NADP	Cofactors, Vitamins, Prosthetic Groups, Pigments	13	7	6	15
Naphtalene_and_antracene_degradation	Peripheral pathways for catabolism of aromatic cor	Metabolism of Aromatic Compounds	6	1	1	1
Neotrehalosadiamine_(NTD)_Biosynthesis_Operon	Aminosugars	Carbohydrates	12	26	8	11
N-heterocyclic_aromatic_compound_degradation	Metabolism of central aromatic intermediates	Metabolism of Aromatic Compounds	1948	1990	13329	1709
NiFe_hydrogenase_maturation	Electron donating reactions	Respiration	771	728	748	762
Nitrate_and_nitrite_ammonification	Unknown	Nitrogen Metabolism	5899	8209	6043	6969
Nitric_oxide_synthase	Unknown	Nitrogen Metabolism	54	89	34	46
Nitrogen_fixation	Unknown	Nitrogen Metabolism	417	479	299	358
Nitrosative_stress	Unknown	Nitrogen Metabolism	2010	2786	1508	1384
N-linked_Glycosylation_in_Bacteria	Protein processing and modification	Protein Metabolism	262	183	150	215

SEED L3	SEED L2	SEED L1	Site S1	Site S2	Site S3	Site S4
Nonhomologous_End-Joining_in_Bacteria	DNA repair	DNA Metabolism	186	195	344	268
Nonribosomal_peptide_synthetases_(NRPS)_in_Frankia_sp._Ccl3	Bacterial cytostatics, differentiation factors and ant	Secondary Metabolism	1	3	1	1
Novel_non-oxidative_pathway_of_Uracil_catabolism	Pyrimidines	Nucleosides and Nucleotides	56	49	47	70
n-Phenylalkanoic_acid_degradation	Peripheral pathways for catabolism of aromatic cor	Metabolism of Aromatic Compounds	249	326	173	139
Nucleoside_triphosphate_pyrophosphohydrolase_MazG	Detoxification	Nucleosides and Nucleotides	45	86	30	99
Nudix_proteins_(nucleoside_triphosphate_hydrolases)	Detoxification	Nucleosides and Nucleotides	223	288	7697	439
NusA-TFII_Cluster	Unknown	Clustering-based subsystems	22	18	16	41
Octadecanoids	Plant Octadecanoids	Secondary Metabolism	2	2	2	5
Omega_peptidases_(EC_3.4.19.-)	Protein degradation	Protein Metabolism	124	64	44	77
One-carbon_metabolism_by_tetrahydropterines	One-carbon Metabolism	Carbohydrates	330	403	215	265
Orphan_regulatory_proteins	Unknown	Regulation and Cell signaling	1004	711	768	927
Osmoprotectant_ABC_transporter_YehZYXW_of_Enterobacteriales	Osmotic stress	Stress Response	29	24	19	57
Osmoregulation	Osmotic stress	Stress Response	2802	2454	1698	1457
Outer_membrane	Gram-Negative cell wall components	Cell Wall and Capsule	561	528	349	361
Oxidative_stress	Oxidative stress	Stress Response	11445	12254	7721	7645
P38_MAP_kinase_pathways	Signal transduction in Eukaryotes	Regulation and Cell signaling	311	201	127	184
PA0057_cluster	Unknown	Clustering-based subsystems	31	25	60	20
p-Aminobenzoyl-Glutamate_Utilization	Folate and pterines	Cofactors, Vitamins, Prosthetic Groups, Pigments	32	12	10	29
Particulate_methane_monooxygenase_(pMMO)	Central carbohydrate metabolism	Carbohydrates	1990	1318	1834	441
p-cymene_degradation	Unknown	Metabolism of Aromatic Compounds	2	2	2	5
Pentose_phosphate_pathway	Central carbohydrate metabolism	Carbohydrates	2318	2379	1588	2145
Peptide_methionine_sulfoxide_reductase	Protein processing and modification	Protein Metabolism	2361	1078	1318	960
Peptidoglycan_Biosynthesis	Unknown	Cell Wall and Capsule	8716	21946	5687	8121
Peptidoglycan_biosynthesis--gjo	Unknown	Cell Wall and Capsule	1154	931	1031	987
Peptidoglycan_Crosslinking_of_Peptide_Stems	Unknown	Cell Wall and Capsule	113	154	45	44
Peptidyl-prolyl_cis-trans_isomerase	Protein folding	Protein Metabolism	1172	566	662	779
Peripheral_Glucose_Catabolism_Pathways	Central carbohydrate metabolism	Carbohydrates	527	177	592	377
Periplasmic_disulfide_interchange	Protein folding	Protein Metabolism	758	923	614	725
Periplasmic_Stress_Response	Periplasmic Stress	Stress Response	3202	2447	2256	1804
Periplasmic-Binding-Protein-Dependent_Transport_System_for_α-Glucosides	ABC transporters	Membrane Transport	1031	1245	581	558
Perosamine_Synthesis_Vibrio	Gram-Negative cell wall components	Cell Wall and Capsule	43	80	62	42
Persister_Cells	Unknown	Dormancy and Sporulation	254	133	75	67
Phage_baseplate_proteins	Phages, Prophages	Phages, Prophages, Transposable elements, Plasmids	6	1	6	6
Phage_capsid_proteins	Bacteriophage structural proteins	Phages, Prophages, Transposable elements	37	51	76	17
Phage_entry_and_exit	Phage Host Interactions	Phages, Prophages, Transposable elements	15	8	16	6
Phage_integration_and_excision	Bacteriophage integration/excision/lysogeny	Phages, Prophages, Transposable elements	27	35	24	46
Phage_packaging_machinery	Phages, Prophages	Phages, Prophages, Transposable elements, Plasmids	22	6	5	5
Phage_shock_protein_(psp)_operon	Unknown	Stress Response	438	312	240	213
Phage_tail_fiber_proteins	Phages, Prophages	Phages, Prophages, Transposable elements, Plasmids	164	319	283	103
Phage_tail_proteins_2	Phages, Prophages	Phages, Prophages, Transposable elements, Plasmids	2	2	2	5
Phd-Doc,_YdcE-YdcD_toxin-antitoxin_(programmed_cell_death)_systems	Programmed Cell Death and Toxin-antitoxin Systen	Regulation and Cell signaling	201	170	158	212
Phenol_hydroxylase	Peripheral pathways for catabolism of aromatic cor	Metabolism of Aromatic Compounds	71	104	125	139
Phenylacetyl-CoA_catabolic_pathway_(core)	Unknown	Metabolism of Aromatic Compounds	363	302	470	420
Phenylalanine_and_Tyrosine_Branches_from_Chorismate	Aromatic amino acids and derivatives	Amino Acids and Derivatives	153	84	147	213
Phenylpropanoid_compound_degradation	Peripheral pathways for catabolism of aromatic cor	Metabolism of Aromatic Compounds	444	481	161	434
Phosphate_metabolism	Unknown	Phosphorus Metabolism	14477	13304	11160	13645
Phosphoenolpyruvate_phosphomutase	Unknown	Phosphorus Metabolism	98	77	143	114

SEED L3	SEED L2	SEED L1	Site S1	Site S2	Site S3	Site S4
Phosphoglycerate_transport_system	Unknown	Membrane Transport	7	6	5	14
Phospholipid_and_Fatty_acid_biosynthesis_related_cluster	Fatty acids	Fatty Acids, Lipids, and Isoprenoids	347	338	195	496
Phosphonate_metabolism	Unknown	Phosphorus Metabolism	36	29	64	29
Phosphorylcholine_incorporation_in_LPS	Capsular and extracellular polysacchrides	Cell Wall and Capsule	11	9	7	59
Photorespiration_(oxidative_C2_cycle)	CO2 fixation	Carbohydrates	992	1081	499	829
Photosystem_I	Electron transport and photophosphorylation	Photosynthesis	13414	2297	14966	28809
Photosystem_II	Electron transport and photophosphorylation	Photosynthesis	40792	20762	#####	#####
Photosystem_II-type_photosynthetic_reaction_center	Electron transport and photophosphorylation	Photosynthesis	325	62	81	50
Phycobilisome	Light-harvesting complexes	Photosynthesis	10790	1078	2288	1321
p-Hydroxybenzoate_degradation	Peripheral pathways for catabolism of aromatic cor	Metabolism of Aromatic Compounds	66	83	48	122
Phytosterol_biosynthesis	Biosynthesis of phenylpropanoids	Secondary Metabolism	4	1	1	4
Plasmid_replication	DNA replication	DNA Metabolism	104	38	31	64
Plasmid-encoded_T-DNA_transfer	Plasmid related functions	Phages, Prophages, Transposable elements, Plasmids	4	3	2	7
Plastoquinone_Biosynthesis	Quinone cofactors	Cofactors, Vitamins, Prosthetic Groups, Pigments	2	8	3	2
Polyadenylation_bacterial	RNA processing and modification	RNA Metabolism	6334	3339	2864	3179
Polyamine_Metabolism	Arginine; urea cycle, polyamines	Amino Acids and Derivatives	5981	5736	4680	4316
Poly-gamma-glutamate_biosynthesis	Glutamine, glutamate, aspartate, asparagine; amm	Amino Acids and Derivatives	9	15	12	10
Polyglycerolphosphate_lipoteichoic_acid_biosynthesis	Gram-Positive cell wall components	Cell Wall and Capsule	7	6	24	5
Polyhydroxybutyrate_metabolism	Unknown	Fatty Acids, Lipids, and Isoprenoids	2376	1792	1444	1682
Polyprenyl_Diphosphate_Biosynthesis	Isoprenoids	Fatty Acids, Lipids, and Isoprenoids	698	527	408	446
polyprenyl_synthesis	Isoprenoids	Fatty Acids, Lipids, and Isoprenoids	6	5	23	4
Polysaccharide_deacetylases	Capsular and extracellular polysacchrides	Cell Wall and Capsule	2	2	10	2
Polyunsaturated_Fatty_Acids_synthesis	Fatty acids	Fatty Acids, Lipids, and Isoprenoids	151	127	446	102
Potassium_homeostasis	Unknown	Potassium metabolism	2411	2560	1913	1828
Predicted_carbohydrate_hydrolases	Glycoside hydrolases	Carbohydrates	248	156	182	103
Predicted_mycobacterial_monooxygenase	Carbohydrates	Clustering-based subsystems	38	80	65	25
Programmed_frameshift	Protein biosynthesis	Protein Metabolism	1268	804	881	836
Proline,_4-hydroxyproline_uptake_and_utilization	Proline and 4-hydroxyproline	Amino Acids and Derivatives	1757	1079	1072	1261
Proline_Synthesis	Proline and 4-hydroxyproline	Amino Acids and Derivatives	349	245	199	229
Propanediol_utilization	Sugar alcohols	Carbohydrates	99	171	165	83
Propionate-CoA_to_Succinate_Module	Organic acids	Carbohydrates	116	275	166	272
Propionyl-CoA_to_Succinyl-CoA_Module	Organic acids	Carbohydrates	992	715	498	615
Proteasome_archaeal	Protein degradation	Protein Metabolism	525	793	532	629
Proteasome_bacterial	Protein degradation	Protein Metabolism	201	97	82	192
Proteasome_eukaryotic	Protein degradation	Protein Metabolism	1748	1191	1079	1197
Proteasome_subunit_alpha_archaeal_cluster	Proteasome related clusters	Clustering-based subsystems	61	108	66	89
Protection_from_Reactive_Oxygen_Species	Oxidative stress	Stress Response	172	85	125	130
Protein_Acetylation_and_Deacetylation_in_Bacteria	Protein processing and modification	Protein Metabolism	50	40	23	15
Protein_chaperones	Protein folding	Protein Metabolism	9130	8207	6806	6916
Protein_degradation	Protein degradation	Protein Metabolism	1214	1143	765	1135
Protein_secretion_by_ABC-type_exporters	Secretion	Protein Metabolism	87	76	72	116
Proteolysis_in_bacteria,_ATP-dependent	Protein degradation	Protein Metabolism	29610	22340	17185	19142
Protocatechuate_branch_of_beta-ketoadipate_pathway	Metabolism of central aromatic intermediates	Metabolism of Aromatic Compounds	85	171	97	61
Proton-dependent_Peptide_Transporters	Uni- Sym- and Antiporters	Membrane Transport	227	176	268	156
Pseudaminic_Acid_Biosynthesis	Capsular and extracellular polysacchrides	Cell Wall and Capsule	67	100	41	78
Pseudomonas_quinolone_signal_PQS	Unknown	Regulation and Cell signaling	20	41	14	11
Pseudouridine_Metabolism	Unknown	Nucleosides and Nucleotides	2	10	2	2

SEED L3	SEED L2	SEED L1	Site S1	Site S2	Site S3	Site S4
Pterin_biosynthesis	Folate and pterines	Cofactors, Vitamins, Prosthetic Groups, Pigments	18	38	35	7
Pterin_carbinolamine_dehydratase	Folate and pterines	Cofactors, Vitamins, Prosthetic Groups, Pigments	23	17	9	42
Pterin_metabolism	Folate and pterines	Cofactors, Vitamins, Prosthetic Groups, Pigments	13	7	6	28
Pterin_metabolism_3	Folate and pterines	Cofactors, Vitamins, Prosthetic Groups, Pigments	1948	1255	853	1116
Purine_conversions	Purines	Nucleosides and Nucleotides	12033	10981	7392	9429
Purine_Utilization	Purines	Nucleosides and Nucleotides	1869	1476	972	1450
Putative_diaminopropionate_ammonia-lyase_cluster	Clustering-based subsystems	Clustering-based subsystems	65	176	83	82
Putative_sugar_ABC_transporter_(ytf_cluster)	Carbohydrates	Clustering-based subsystems	67	111	68	39
Putative_TldE-TldD_proteolytic_complex	Protein degradation	Protein Metabolism	937	617	375	467
Putrescine_utilization_pathways	Arginine; urea cycle, polyamines	Amino Acids and Derivatives	826	625	625	694
pVir_Plasmid_of_Campylobacter	Protein and nucleoprotein secretion system, Type I	Membrane Transport	7	6	5	14
Pyridoxin_(Vitamin_B6)_Biosynthesis	Pyridoxine	Cofactors, Vitamins, Prosthetic Groups, Pigments	6424	4570	4308	4122
pyrimidine_conversions	Pyrimidines	Nucleosides and Nucleotides	4606	3707	2395	2925
Pyrimidine_utilization	Pyrimidines	Nucleosides and Nucleotides	566	335	228	257
Pyrroloquinoline_Quinone_biosynthesis	Quinone cofactors	Cofactors, Vitamins, Prosthetic Groups, Pigments	208	359	161	621
Pyruvate:ferredoxin_oxidoreductase	Central carbohydrate metabolism	Carbohydrates	1947	2553	1913	2052
Pyruvate_Alanine_Serine_Interconversions	Central carbohydrate metabolism	Carbohydrates	1407	1303	1048	1188
Pyruvate_metabolism_I:_anaplerotic_reactions,_PEP	Central carbohydrate metabolism	Carbohydrates	8945	9060	6447	7332
Pyruvate_metabolism_II:_acetyl-CoA,_acetogenesis_from_pyruvate	Central carbohydrate metabolism	Carbohydrates	6084	6465	5331	4716
Queuosine-Archaeosine_Biosynthesis	RNA processing and modification	RNA Metabolism	3732	2038	2187	2207
Quinolinic_acid_and_its_derivatives	Biologically active compounds in metazoan cell def	Secondary Metabolism	1	1	20	1
Quinone_oxidoreductase_family	Unknown	Respiration	308	368	358	376
Quorum_sensing_in_Yersinia	Quorum sensing and biofilm formation	Regulation and Cell signaling	10	8	10	16
Rcs_phosphorelay_signal_transduction_pathway	Unknown	Regulation and Cell signaling	11	11	20	7
Recycling_of_Peptidoglycan_Amino_Acids	Unknown	Cell Wall and Capsule	363	375	296	295
Recycling_of_Peptidoglycan_Amino_Sugars	Unknown	Cell Wall and Capsule	262	170	190	258
Redox-dependent_regulation_of_nucleus_processes	Oxidative stress	Stress Response	2495	3126	3294	2530
Reductive_Dechlorination	Unknown	Respiration	16	13	10	31
Regulation_of_Oxidative_Stress_Response	Oxidative stress	Stress Response	130	124	119	174
Regulatory_Intramembrane_Proteolysis_Pathways	Proteolytic pathway	Regulation and Cell signaling	1239	866	1450	823
Release_of_Dimethyl_Sulfide_(DMS)_from_Dimethylsulfoniopropionate_(DMSP)	Unknown	Sulfur Metabolism	18	13	6	6
Resistance_to_chromium_compounds	Resistance to antibiotics and toxic compounds	Virulence, Disease and Defense	32	26	63	21
Resistance_to_fluoroquinolones	Resistance to antibiotics and toxic compounds	Virulence, Disease and Defense	6954	5911	4632	5877
Resistance_to_Vancomycin	Resistance to antibiotics and toxic compounds	Virulence, Disease and Defense	49	69	72	35
Respiratory_Complex_I	Electron donating reactions	Respiration	13648	11640	9296	13257
Respiratory_dehydrogenases_1	Electron donating reactions	Respiration	6600	4812	3964	3306
Restriction-Modification_System	Unknown	DNA Metabolism	327	214	330	262
Rhamnose_containing_glycans	Capsular and extracellular polysacchrides	Cell Wall and Capsule	4018	3442	2621	3764
Riboflavin,_FMN_and_FAD_metabolism	Riboflavin, FMN, FAD	Cofactors, Vitamins, Prosthetic Groups, Pigments	1025	879	720	682
riboflavin_to_FAD	Riboflavin, FMN, FAD	Cofactors, Vitamins, Prosthetic Groups, Pigments	25	4	6	3
Ribonuclease_H	RNA processing and modification	RNA Metabolism	192	129	149	273
Ribonucleotide_reduction	Unknown	Nucleosides and Nucleotides	6414	5552	4280	4654
Ribosomal_protein_S12p_Asp_methylthiotransferase	Protein processing and modification	Protein Metabolism	136	54	59	47
Ribosomal_protein_S5p_acylation	Protein processing and modification	Protein Metabolism	4845	4120	2869	2692
Ribosome_activity_modulation	Protein biosynthesis	Protein Metabolism	848	493	299	302
Ribosome_biogenesis_archaeal	RNA processing and modification	RNA Metabolism	2	2	2	6
Ribosome_biogenesis_bacterial	Protein biosynthesis	Protein Metabolism	5091	4188	2692	3662

SEED L3	SEED L2	SEED L1	Site S1	Site S2	Site S3	Site S4
Ribosome_LSU_bacterial	Protein biosynthesis	Protein Metabolism	92443	73657	60781	66920
Ribosome_LSU_chloroplast	Protein biosynthesis	Protein Metabolism	9	7	17	10
Ribosome_LSU_eukaryotic_and_archaeal	Protein biosynthesis	Protein Metabolism	463	375	262	304
Ribosome_LSU_mitochondrial	Protein biosynthesis	Protein Metabolism	4	7	2	4
Ribosome_SSU_bacterial	Protein biosynthesis	Protein Metabolism	81257	63344	73438	57930
Ribosome_SSU_chloroplast	Protein biosynthesis	Protein Metabolism	33	25	30	29
Ribosome_SSU_eukaryotic_and_archaeal	Protein biosynthesis	Protein Metabolism	852	573	589	526
Ribosome_SSU_mitochondrial	Protein biosynthesis	Protein Metabolism	51	85	11	25
RNA_3'-terminal_phosphate_cyclase	RNA processing and modification	RNA Metabolism	181	209	140	112
RNA_polymerase_archaeal	Transcription	RNA Metabolism	50	76	38	77
RNA_polymerase_archaeal_initiation_factors	Transcription	RNA Metabolism	26	7	7	6
RNA_polymerase_bacterial	Transcription	RNA Metabolism	52017	43939	33570	43521
RNA_polymerase_chloroplast	Transcription	RNA Metabolism	10	8	16	21
RNA_polymerase_I	Transcription	RNA Metabolism	249	52	35	36
RNA_polymerase_II	Transcription	RNA Metabolism	543	192	292	216
RNA_polymerase_II_initiation_factors	Transcription	RNA Metabolism	76	59	108	62
RNA_polymerase_III	Transcription	RNA Metabolism	406	102	274	137
RNA_polymerase_III_initiation_factors	Transcription	RNA Metabolism	27	4	10	14
RNA_processing_and_degradation,_bacterial	RNA processing and modification	RNA Metabolism	2343	1880	1716	2023
RNA_pseudouridine_syntheses	RNA processing and modification	RNA Metabolism	67	74	51	101
Rrf2_family_transcriptional_regulators	Transcription	RNA Metabolism	949	329	288	236
rRNA_modification_Archaea	RNA processing and modification	RNA Metabolism	88	35	51	57
rRNA_modification_Bacteria	RNA processing and modification	RNA Metabolism	121	76	33	83
Rubrerythrin	Oxidative stress	Stress Response	946	1412	762	591
RuvABC_plus_a_hypothetical	DNA recombination	DNA Metabolism	1377	819	734	819
Salicylate_and_gentisate_catabolism	Metabolism of central aromatic intermediates	Metabolism of Aromatic Compounds	669	410	416	407
Salicylate_ester_degradation	Peripheral pathways for catabolism of aromatic cor	Metabolism of Aromatic Compounds	13	90	27	12
Salmochelin-mediated_Iron_Acquisition	Siderophores	Iron acquisition and metabolism	21	17	38	20
Selenocysteine_metabolism	Selenoproteins	Protein Metabolism	875	501	486	472
Selenoprotein_O	Selenoproteins	Protein Metabolism	260	209	130	168
SeqA_and_Co-occurring_Genes	Chromosome Replication	Clustering-based subsystems	98	37	26	44
Serine_Biosynthesis	Alanine, serine, and glycine	Amino Acids and Derivatives	33	40	31	69
Serine_endopeptidase_(EC_3.4.21.-)	Protein degradation	Protein Metabolism	474	291	475	362
Serine-glyoxylate_cycle	One-carbon Metabolism	Carbohydrates	21212	25180	14459	18062
Serotype_determining_Capsular_polysaccharide_biosynthesis_in_Staphylococcus	Capsular and extracellular polysacchrides	Cell Wall and Capsule	6	5	12	4
Sialic_Acid_Metabolism	Capsular and extracellular polysacchrides	Cell Wall and Capsule	2392	2239	1400	2037
Siderophore_Achromobactin	Siderophores	Iron acquisition and metabolism	10	24	6	6
Siderophore_Aerobactin	Siderophores	Iron acquisition and metabolism	1	1	1	2
Siderophore_assembly_kit	Siderophores	Iron acquisition and metabolism	92	62	62	91
Siderophore_Desferrioxamine_E	Siderophores	Iron acquisition and metabolism	16	5	4	9
Siderophore_Enterobactin	Siderophores	Iron acquisition and metabolism	37	87	24	30
Siderophore_pyochelin	Siderophores	Iron acquisition and metabolism	10	8	5	6
Siderophore_Pyoverdine	Siderophores	Iron acquisition and metabolism	601	270	130	518
Siderophore_Yersiniabactin_Biosynthesis	Siderophores	Iron acquisition and metabolism	5	12	3	5
SigmaB_stress_responce_regulation	Unknown	Stress Response	754	1821	821	1439
Signal_peptidase	Protein processing and modification	Protein Metabolism	622	585	429	891
Small_acid-soluble_spore_proteins	Spore DNA protection	Dormancy and Sporulation	31	25	20	59

SEED L3	SEED L2	SEED L1	Site S1	Site S2	Site S3	Site S4
Soluble_cytochromes_and_functionally_related_electron_carriers	Unknown	Respiration	1930	1191	1008	1062
Soluble_methane_monooxygenase_(sMMO)	Central carbohydrate metabolism	Carbohydrates	477	285	453	142
Sphingolipid_biosynthesis	Phospholipids	Fatty Acids, Lipids, and Isoprenoids	1	7	1	1
Spliceosome	RNA processing and modification	RNA Metabolism	423	241	51	161
Spore_Coat	Unknown	Clustering-based subsystems	140	101	92	32
Spore_germination	Unknown	Dormancy and Sporulation	12	6	26	13
Spore_pigment_biosynthetic_cluster_in_Actinomycetes	Unknown	Dormancy and Sporulation	34	28	23	67
Sporulation_gene_orphans	Unknown	Dormancy and Sporulation	98	106	64	52
Sporulation-associated_proteins_with_broader_functions	Unknown	Dormancy and Sporulation	324	402	412	507
Sporulation-related_Hypotheticals	Clustering-based subsystems	Clustering-based subsystems	101	75	32	131
SpoVS_protein_family	Unknown	Dormancy and Sporulation	25	20	16	52
Staphylococcal_phi-Mu50B-like_prophages	Phages, Prophages	Phages, Prophages, Transposable elements, Plasmids	2816	2810	2077	1999
Staphylococcus_aureus_hypothetical_repetitive_gene_loci	Unknown	Clustering-based subsystems	16	13	31	10
Streptococcal_Hyaluronic_Acid_Capsule	Capsular and extracellular polysacchrides	Cell Wall and Capsule	2350	1880	2715	2061
Streptococcus_pyogenes_recombinatorial_zone	Adhesion	Virulence, Disease and Defense	16	13	31	10
Streptococcus_pyogenes_Virulome	Unknown	Virulence, Disease and Defense	50	21	17	61
Streptolysin_S_Biosynthesis_and_Transport	Toxins and superantigens	Virulence, Disease and Defense	86	37	23	40
Streptothricin_resistance	Resistance to antibiotics and toxic compounds	Virulence, Disease and Defense	31	25	60	20
Stringent_Response,_(p)ppGpp_metabolism	Unknown	Regulation and Cell signaling	154	106	161	185
Succinate_dehydrogenase	Electron donating reactions	Respiration	1130	1070	815	1055
Sucrose_utilization	Di- and oligosaccharides	Carbohydrates	300	208	165	129
Sugar_utilization_in_Thermotogales	Unknown	Carbohydrates	83	89	59	152
Sulfatases_and_sulfatase_modifying_factor_1	Sulfatases and sulfatase modifying factor 1 (and a h	Clustering-based subsystems	519	445	351	392
Sulfate_reduction-associated_complexes	Unknown	Sulfur Metabolism	342	766	266	341
Sulfur_oxidation	Unknown	Sulfur Metabolism	1781	2321	6092	1225
Symbiotic_colonization_and_sigma-dependent_biofilm_formation_gene_cluster	Quorum sensing and biofilm formation	Regulation and Cell signaling	185	82	99	113
Synechocystis_experimental	Plant-Prokaryote DOE project	Miscellaneous	112	80	57	88
Synthesis_of_osmoregulated_periplasmic_glucans	Osmotic stress	Stress Response	785	822	498	656
T7-like_cyanophage_core_proteins	Phage family-specific subsystems	Phages, Prophages, Transposable elements, Plasmids	28	13	18	64
Taurine_Utilization	Organic sulfur assimilation	Sulfur Metabolism	162	132	156	154
TCA_Cycle	Central carbohydrate metabolism	Carbohydrates	6713	7570	5216	5129
Teichoic_and_lipoteichoic_acids_biosynthesis	Gram-Positive cell wall components	Cell Wall and Capsule	211	166	195	131
Teichuronic_acid_biosynthesis	Gram-Positive cell wall components	Cell Wall and Capsule	215	139	213	211
Tellurite_resistance:_Chromosomal_determinants	Detoxification	Stress Response	1	3	1	1
Terminal_cytochrome_C_oxidases	Electron accepting reactions	Respiration	12781	12258	8699	12827
Terminal_cytochrome_d_ubiquinol_oxidases	Electron accepting reactions	Respiration	283	581	205	462
Terminal_cytochrome_O_ubiquinol_oxidase	Electron accepting reactions	Respiration	63	145	30	45
Terminal_cytochrome_oxidases	Electron accepting reactions	Respiration	223	18	14	40
Tetrathionate_respiration	Electron accepting reactions	Respiration	76	146	53	73
The_Chv_regulatory_system_of_Alphaproteobacteria	Unknown	Regulation and Cell signaling	146	88	150	126
The_mdtABCD_multidrug_resistance_cluster	Resistance to antibiotics and toxic compounds	Virulence, Disease and Defense	107	135	123	118
Thermosome,_archaeal	Protein folding	Protein Metabolism	315	210	261	215
Thiamin_biosynthesis	Unknown	Cofactors, Vitamins, Prosthetic Groups, Pigments	1059	860	535	572
Thioredoxin-disulfide_reductase	Unknown	Sulfur Metabolism	5830	6555	3997	2834
Threonine_anaerobic_catabolism_gene_cluster	Lysine, threonine, methionine, and cysteine	Amino Acids and Derivatives	5	28	3	3
Threonine_and_Homoserine_Biosynthesis	Lysine, threonine, methionine, and cysteine	Amino Acids and Derivatives	3609	2931	2513	2640
Threonine_degradation	Lysine, threonine, methionine, and cysteine	Amino Acids and Derivatives	341	235	293	162

SEED L3	SEED L2	SEED L1	Site S1	Site S2	Site S3	Site S4
Tocopherol_Biosynthesis	Quinone cofactors	Cofactors, Vitamins, Prosthetic Groups, Pigments	4	3	14	2
Tolerance_to_colicin_E2	Bacteriocins, ribosomally synthesized antibacterial	Virulence, Disease and Defense	61	77	27	35
Toluene_4-monooxygenase_(T4MO)	Unknown	Metabolism of Aromatic Compounds	56	47	59	17
Toluene_degradation	Peripheral pathways for catabolism of aromatic cor	Metabolism of Aromatic Compounds	67	82	44	47
Ton_and_Tol_transport_systems	Unknown	Membrane Transport	13812	11303	9544	10933
Toxin-antitoxin_systems_(other_than_RelBE_and_MazEF)	Programmed Cell Death and Toxin-antitoxin System	Regulation and Cell signaling	407	345	196	285
Transcription_factors_bacterial	Transcription	RNA Metabolism	13453	10957	9860	10652
Transcription_factors_cyanobacterial_RpoD-like_sigma_factors	Transcription	RNA Metabolism	99	29	28	34
Transcription_initiation,_bacterial_sigma_factors	Transcription	RNA Metabolism	11853	9923	9077	9812
Translation_elongation_factor_G_family	Protein biosynthesis	Protein Metabolism	173	96	83	153
Translation_elongation_factors_bacterial	Protein biosynthesis	Protein Metabolism	67396	58869	40004	46741
Translation_elongation_factors_eukaryotic_and_archaeal	Protein biosynthesis	Protein Metabolism	8356	3290	3621	2220
Translation_initiation_factors_bacterial	Protein biosynthesis	Protein Metabolism	6592	5779	3758	5126
Translation_initiation_factors_eukaryotic_and_archaeal	Protein biosynthesis	Protein Metabolism	55	36	30	116
Translation_termination_factors_bacterial	Protein biosynthesis	Protein Metabolism	3222	2147	1510	2016
Transport_of_Iron	Unknown	Iron acquisition and metabolism	156	107	187	175
Transport_of_Manganese	Unknown	Membrane Transport	142	134	127	152
Transport_of_Molybdenum	Unknown	Membrane Transport	2	2	2	5
Transport_of_Nickel_and_Cobalt	Unknown	Membrane Transport	254	236	245	154
Transport_of_Zinc	Unknown	Membrane Transport	374	165	216	205
Trans-translation_by_stalled_ribosomes	Protein biosynthesis	Protein Metabolism	105	116	117	78
Trehalose_Biosynthesis	Di- and oligosaccharides	Carbohydrates	726	639	545	445
Trehalose_Uptake_and_Utilization	Di- and oligosaccharides	Carbohydrates	250	542	269	165
Triacylglycerol_metabolism	Triacylglycerols	Fatty Acids, Lipids, and Isoprenoids	77	62	92	108
Tricarballylate_Utilization	Organic acids	Carbohydrates	123	49	40	79
trimethylamine_N-oxide_(TMAO)_reductase	Electron accepting reactions	Respiration	221	177	198	228
tRNA_aminoacylation,_Ala	Protein biosynthesis	Protein Metabolism	1326	1674	971	1329
tRNA_aminoacylation,_Arg	Protein biosynthesis	Protein Metabolism	595	281	361	482
tRNA_aminoacylation,_Asp_and_Asn	Protein biosynthesis	Protein Metabolism	4673	3586	3139	3203
tRNA_aminoacylation,_Cys	Protein biosynthesis	Protein Metabolism	698	628	7669	652
tRNA_aminoacylation,_Glu_and_Gln	Protein biosynthesis	Protein Metabolism	1600	2666	1113	1367
tRNA_aminoacylation,_Gly	Protein biosynthesis	Protein Metabolism	1279	1200	972	1119
tRNA_aminoacylation,_His	Protein biosynthesis	Protein Metabolism	245	246	162	314
tRNA_aminoacylation,_Ile	Protein biosynthesis	Protein Metabolism	2105	1511	994	1120
tRNA_aminoacylation,_Leu	Protein biosynthesis	Protein Metabolism	1998	1477	1261	1799
tRNA_aminoacylation,_Lys	Protein biosynthesis	Protein Metabolism	1353	1145	1072	979
tRNA_aminoacylation,_Met	Protein biosynthesis	Protein Metabolism	1057	781	643	733
tRNA_aminoacylation,_Phe	Protein biosynthesis	Protein Metabolism	2196	1740	1260	2031
tRNA_aminoacylation,_Pro	Protein biosynthesis	Protein Metabolism	1619	1231	986	1062
tRNA_aminoacylation,_Ser	Protein biosynthesis	Protein Metabolism	696	629	492	616
tRNA_aminoacylation,_Thr	Protein biosynthesis	Protein Metabolism	1986	2074	1656	2093
tRNA_aminoacylation,_Trp	Protein biosynthesis	Protein Metabolism	751	561	498	669
tRNA_aminoacylation,_Tyr	Protein biosynthesis	Protein Metabolism	533	533	371	512
tRNA_aminoacylation,_Val	Protein biosynthesis	Protein Metabolism	1564	1902	1203	1302
tRNA_modification_Archaea	RNA processing and modification	RNA Metabolism	1914	2053	1596	1437
tRNA_modification_Bacteria	RNA processing and modification	RNA Metabolism	297	335	164	201
tRNA_modification_yeast_cytoplasmic	RNA processing and modification	RNA Metabolism	4	3	10	4

SEED L3	SEED L2	SEED L1	Site S1	Site S2	Site S3	Site S4
tRNA_nucleotidyltransferase	RNA processing and modification	RNA Metabolism	292	325	166	162
tRNA_processing	RNA processing and modification	RNA Metabolism	1310	646	683	739
tRNA-dependent_amino_acid_transfers	Unknown	Cell Wall and Capsule	2	2	2	5
tRNAs	Protein biosynthesis	Protein Metabolism	167	73	88	90
Tryptophan_catabolism	Aromatic amino acids and derivatives	Amino Acids and Derivatives	12	12	11	6
Tryptophan_synthesis	Aromatic amino acids and derivatives	Amino Acids and Derivatives	2055	1829	1362	1712
Twin-arginine_translocation_system	Protein translocation across cytoplasmic membran	Membrane Transport	964	846	503	1036
Two_cell_division_clusters_relating_to_chromosome_partitioning	Unknown	Cell Division and Cell Cycle	2502	2035	1181	1860
Two-component_regulatory_systems_in_Campylobacter	Unknown	Regulation and Cell signaling	184	69	103	63
Two-component_Response_Regulator_of_Virulence_ResDE	Regulation of virulence	Regulation and Cell signaling	34	45	29	19
Type_4_secretion_and_conjugative_transfer	Type III, Type IV, Type VI, ESAT secretion systems	Virulence	17	6	11	84
Type_I_Restriction-Modification	Unknown	DNA Metabolism	1422	1435	976	972
Type_III_secretion_system_orphans	Protein secretion system, Type III	Membrane Transport	43	34	110	199
Type_III_secretion_systems,_extended	Type III secretion system, extended	Clustering-based subsystems	4	1	1	1
Type_IV_pilus	Protein and nucleoprotein secretion system, Type I	Membrane Transport	6312	6341	5108	6404
Type_VI_secretion_systems	Protein secretion system, Type VI	Membrane Transport	3071	1734	1726	2129
Ubiquinone_Biosynthesis	Quinone cofactors	Cofactors, Vitamins, Prosthetic Groups, Pigments	1737	1231	1078	1182
Ubiquinone_Menaquinone-cytochrome_c_reductase_complexes	Electron accepting reactions	Respiration	2633	2841	1981	2722
UDP-N-acetylmuramate_from_Fructose-6-phosphate_Biosynthesis	Unknown	Cell Wall and Capsule	500	361	293	383
Universal_GTPases	Protein biosynthesis	Protein Metabolism	10423	14764	7027	8427
Universal_stress_protein_family	Unknown	Stress Response	346	351	315	409
Unknown	Unknown	Unknown	97315	79654	62807	70385
Unknown_carbohydrate_utilization_(_cluster_Ydj_)	Unknown	Carbohydrates	31	58	22	20
Unknown_carbohydrate_utilization_(_cluster_Yeg_)	Unknown	Carbohydrates	115	91	35	35
Unknown_oligosaccharide_utilization_Sde_1396	Di- and oligosaccharides	Carbohydrates	22	20	18	16
Unspecified_monosaccharide_transport_cluster	Monosaccharides	Clustering-based subsystems	203	235	285	242
Uptake_of_selenate_and_selenite	Detoxification	Stress Response	1813	955	769	911
Uracil-DNA_glycosylase	DNA repair	DNA Metabolism	299	142	217	234
Urea_decomposition	Arginine; urea cycle, polyamines	Amino Acids and Derivatives	2400	1942	1918	1724
USS-DB-1	Unknown	Clustering-based subsystems	10	8	6	19
USS-DB-2	Unknown	Clustering-based subsystems	2	6	3	2
USS-DB-4	Unknown	Clustering-based subsystems	1	1	1	2
USS-DB-6	Unknown	Clustering-based subsystems	126	96	43	51
USS-DB-7	Unknown	Clustering-based subsystems	4340	3385	3291	3047
Utilization_of_glutathione_as_a_sulphur_source	Organic sulfur assimilation	Sulfur Metabolism	206	250	198	290
Valine_degradation	Branched-chain amino acids	Amino Acids and Derivatives	2676	2994	1662	2148
VC0266	Unknown	Carbohydrates	54	43	76	74
Vibrio_Core_Oligosaccharide_Biosynthesis	Gram-Negative cell wall components	Cell Wall and Capsule	36	76	121	21
Vibrio_Polysaccharide_(VPS)_Biosynthesis	Capsular and extracellular polysacchrides	Cell Wall and Capsule	76	37	27	70
VieSAB_signal_transduction_system_of_Vibrio	Regulation of virulence	Regulation and Cell signaling	25	18	29	11
V-Type_ATP_synthase	ATP synthases	Respiration	728	544	571	529
WhiB_and_WhiB-type_regulatory_proteins_	Unknown	Regulation and Cell signaling	77	72	43	20
Widespread_colonization_island	Protein secretion system, Type II	Membrane Transport	2062	1764	1598	1785
Wyeosine-MimG_Biosynthesis	RNA processing and modification	RNA Metabolism	72	60	10	10
Xanthosine_utilization_(xap_region)	Purines	Nucleosides and Nucleotides	1	1	4	1
Xyloglucan_Utilization	Polysaccharides	Carbohydrates	2	2	2	8
Xylose_utilization	Monosaccharides	Carbohydrates	1205	1073	959	1179

SEED L3	SEED L2	SEED L1	Site S1	Site S2	Site S3	Site S4
YaaA	Unknown	Miscellaneous	60	147	80	39
YbbK	Unknown	Miscellaneous	1248	962	741	860
YcfH	Unknown	DNA Metabolism	253	95	158	84
YeiH	Lysine, threonine, methionine, and cysteine	Clustering-based subsystems	65	50	121	40
Yfa_cluster	Unknown	Clustering-based subsystems	20	45	14	13
YjeE	Unknown	Cell Wall and Capsule	288	137	215	164
YrdC-YciO	RNA processing and modification	RNA Metabolism	141	121	86	108
Zinc_regulated_enzymes	Unknown	Regulation and Cell signaling	1896	1351	884	1287
Zinc_resistance	Resistance to antibiotics and toxic compounds	Virulence, Disease and Defense	1162	1321	915	1330
ZZ_gjo_need_homes	Unknown	Miscellaneous	2904	2434	2009	2430
