# Supplementary material for: Light Penetration, Dissolved Oxygen, and Riparian Zone Extent Are Associated with the Structure, Gene Expression, and Lipid Production of Riverbed Microbial Communities
Source: Microorganisms. 2026 Oct 21;14(10):2406. doi: 10.3390/microorganisms14102406 (PMC13707542; doi:10.3390/microorganisms14102406)
Supplement: Supplementary file 1 [file microorganisms-14-02406-s001.zip › Supplementary Table S3. Cumulative table of measured fatty acids.pdf]

FA species	Type	Kingdom	Site S1	Site S2	Site S3	Site S4
14:0	C14-C18 SFA	General	69.8	11.5	15.2	22.7
15:0	C14-C18 SFA	General	17.3	4.3	4.2	6.2
15:1w7	BacFA	Prokaryotes	8.6	0.6	1.4	2.5
16:0	C14-C18 SFA	General	511.3	130.1	139.2	203.1
16:1w5	C16 MUFA	Fungi	51.2	8.9	13.6	44.9
16:1w6	C16 MUFA	Prokaryotes	0.0	0.0	0.0	0.0
16:1w7	C16 MUFA	Prokaryotes	326.6	24.0	62.4	127.9
16:1w8	C16 MUFA	Prokaryotes	0.0	0.0	0.0	0.0
16:1w9	C16 MUFA	Prokaryotes	0.0	0.0	0.0	0.0
16:2w4	C16 w-4	Algae	0.0	0.0	0.0	0.0
16:2w6	C16 w-6	Algae	0.0	0.0	0.0	0.0
16:2w7	C16 w-7	Prokaryotes	0.0	0.0	0.0	0.0
16:3w3	C16 w-3	Algae	0.0	0.0	0.0	0.0
16:3w4	C16 w-4	Unknown	0.0	0.0	0.0	0.0
16:3w6	C16 w-6	Unknown	0.0	0.0	0.0	0.0
16:4w1	C16 w-1	Unknown	0.0	0.0	0.0	0.0
16:4w3	C16 w-3	Algae	0.0	0.0	0.0	0.0
17:0	BacFA	General	22.1	3.8	3.4	5.1
18:0	C14-C18 SFA	General	144.8	83.5	52.9	68.6
18:1w7	C18 MUFA	Prokaryotes	159.9	19.4	29.2	51.8
18:1w9	C18 MUFA	Prokaryotes	105.4	25.6	28.0	35.4
18:2w6	C18 MUFA	Fungi	65.8	15.5	16.5	20.2
18:3w3	C18 MUFA	Algae	56.6	14.4	5.6	7.6
18:3w6	C18 MUFA	Prokaryotes	0.0	0.0	0.0	0.0
18:4w3	C18 MUFA	Algae	15.8	1.1	5.1	7.9
18:4w4	C18 MUFA	Unknown	0.0	0.0	0.0	0.0
18:5w3	C18 MUFA	Unknown	0.0	0.0	0.0	0.0
20:0	C20-C26 SFA	General	8.6	5.5	3.6	4.7
20:1w7	C20-C22 MUFA	Unknown	0.0	0.0	0.0	0.0
20:1w9	C20-C22 MUFA	Prokaryotes	15.3	2.3	2.3	3.9
20:2w6	C20 w-6	Algae	0.0	0.0	0.0	0.0
20:3w3	C20 w-3	Algae	0.0	0.0	0.0	0.0
20:3w6	C20 w-6	Algae	0.0	0.0	0.0	0.0
20:4w3	C20 w-3	Unknown	0.0	0.0	0.0	0.0
20:4w6	C20 w-6	Algae	52.9	6.4	12.8	13.3
20:5w3	C20 w-3	Algae	103.9	15.9	68.7	82.3
22:0	C20-C26 SFA	Unknown	11.0	9.2	6.0	10.4
22:1w7	C20-C22 MUFA	Unknown	0.0	0.0	0.0	0.0
22:1w9	C20-C22 MUFA	Prokaryotes	42.0	14.0	12.0	12.1
22:4w6	C22 w-6	Algae	0.0	0.0	0.0	0.0
22:5w3	C22 w-3	Algae	0.0	0.0	0.0	0.0
22:5w6	C22 w-6	Algae	0.0	0.0	0.0	0.0
22:6w3	C22 w-3	Algae	21.9	1.5	8.4	10.1
24:0	C20-C26 SFA	General	11.1	13.1	7.2	11.4
26:0	C20-C26 SFA	Unknown	2.6	5.7	2.9	4.0
a-15:0	BacFA	Prokaryotes	27.8	4.0	6.1	10.8
a-17:0	BacFA	Prokaryotes	11.5	1.9	2.2	4.3
i-14:0	BacFA	Prokaryotes	10.5	1.3	2.1	2.7
i-15:0	BacFA	Prokaryotes	52.2	6.2	9.5	17.0
i-16:0	BacFA	Prokaryotes	17.2	2.8	3.3	5.5
i-17:0	BacFA	Prokaryotes	22.3	3.0	3.8	6.9
